# Targeted Analysis of Mitochondrial Protein Conformations and Interactions by Endogenous ROS‐Triggered Cross‐Linker Release

**DOI:** 10.1002/advs.202408462

**Published:** 2024-10-30

**Authors:** Wen Zhou, Yuwan Chen, Wenxin Fu, Xinwei Li, Yufei Xia, Qun Zhao, Baofeng Zhao, Yukui Zhang, Kaiguang Yang, Lihua Zhang

**Affiliations:** ^1^ State Key Laboratory of Medical Proteomics National Chromatographic R. & A. Center CAS Key Laboratory of Separation Science for Analytical Chemistry Dalian Institute of Chemical Physics Chinese Academy of Sciences 457 Zhongshan Road Dalian 116023 China; ^2^ University of Chinese Academy of Sciences Beijing 100049 China; ^3^ Research Center for Analytical Sciences Northeastern University Shenyang 110819 China; ^4^ School of Chemistry Dalian University of Technology Dalian 116024 China; ^5^ State Key Laboratory of Biochemical Engineering Institute of Process Engineering Chinese Academy of Sciences Beijing 100190 China

**Keywords:** cross‐linking mass spectrometry, mitochondria, nanoparticles, protein complexes, reactive oxygen species‐responsive

## Abstract

The study of in situ conformations and interactions of mitochondrial proteins plays a crucial role in understanding their biological functions. Current chemical cross‐linking mass spectrometry (CX‐MS) has difficulty in achieving in‐depth analysis of mitochondrial proteins for cells without genetic modification. Herein, this work develops the reactive oxygen species (ROS)‐responsive cross‐linker delivery nanoparticles (R‐CDNP) targeting mitochondria. R‐CDNP contains mitochondria‐targeting module triphenylphosphine, ROS‐responsive module thioketal, loading module poly(lactic‐co‐glycolic acid) (PLGA), and polyethylene glycol (PEG), and cross‐linker module disuccinimidyl suberate (DSS). After targeting mitochondria, ROS‐triggered cross‐linker release improves the cross‐linking coverage of mitochondria in situ. In total, this work identifies 2103 cross‐linked sites of 572 mitochondrial proteins in HepG2 cells. 1718 intra‐links reveal dynamic conformations involving chaperones with ATP‐dependent conformation cycles, and 385 inter‐links reveal dynamic interactions involving OXPHOS complexes and 27 pairs of possible potential interactions. These results signify that R‐CDNP can achieve dynamic conformation and interaction analysis of mitochondrial proteins in living cells, thereby contributing to a better understanding of their biological functions.

## Graphical abstract

Endogenous ROS‐responsive cross‐linker delivery nanoparticles (R‐CDNP) are fabricated for the triggered releasement of cross‐linkers at mitochondria in intact living cells. Without genetically modifying cells or isolating mitochondria, R‐CDNP can capture native mitochondrial protein information, further achieving in situ analysis of protein conformations and interactions with the integration of chemical cross‐linking mass spectrometry.

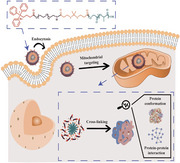

## 1 Introduction

As the cellular powerhouse, mitochondria are important integrators of intermediary metabolism in cellular metabolic pathways,^[^ ^1^ ^]^ such as oxidative phosphorylation (OXPHOS) and tricarboxylic acid (TCA) cycle. Apart from bioenergetics and biosynthesis, mitochondria also participate in cell signaling.^[^ ^2^, ^3^, ^4^ ^]^ Studies have revealed that the disturbances of mitochondrial protein function cause a variety of diseases,^[^ ^5^, ^6^ ^]^ including metabolic disorders and neurological disorders. Protein function largely depends on 3D structures and protein‐protein interactions (PPIs).^[^ ^7^ ^]^ The investigation of mitochondrial protein conformations and PPIs comprehensively can provide insight into protein function and regulation mechanism, which can help elucidate the mechanism of mitochondrial dysfunction and further provide appealing therapeutic targets for mitochondrial diseases.^[^ ^3^, ^5^, ^8^, ^9^ ^]^

Chemical cross‐linking mass spectrometry (CX‐MS) has attracted increasing attention due to the ability to simultaneously perform large‐scale conformation and PPI analysis of complex samples.^[^ ^10^, ^11^, ^12^ ^]^ Current approaches isolated mitochondria from cells or tissues and then used cross‐linkers, such as BDP‐NHP and DSS, for cross‐linking.^[^ ^6^, ^13^, ^14^, ^15^ ^]^ These methods retained the integrity of mitochondria and achieved the mitochondrial protein analysis. However, the purification of mitochondria isolated from the native physiological microenvironment in living cells is insufficient, affecting the accuracy and coverage of the results. In addition, stripping mitochondria might cause irreversible perturbation, which may result in the alteration or loss of protein conformations and PPIs within the mitochondria.

Recently, our research group has developed the in vivo method based on targeted cross‐linker delivery and successfully realized the protein analysis of the mitochondria in HepG2 cells and the chloroplasts in living Chlamydomonas reinhardtii cells.^[^ ^16^, ^17^ ^]^ The method first enables in situ cross‐linking of targeted subcellular organelles in living cells. Nevertheless, the limited cross‐linker release rate at the target sites results in low cross‐linking coverage, so there is an urgent need to solve the release problem.

Since 90% of intracellular ROS are produced by the mitochondrial respiratory chain,^[^ ^18^, ^19^ ^]^ the ROS concentration in mitochondria is high. For example, the ROS concentration of tumor cells reaches up to 100 µM due to abnormal proliferation and increased metabolic activity.^[^ ^20^, ^21^, ^22^ ^]^ Therefore, we utilized mitochondrial ROS to achieve ROS‐triggered cross‐linker release from targeted nanoparticles to ensure high cross‐linking coverage under the premise of low perturbation of living cells.

**Figure 1:**
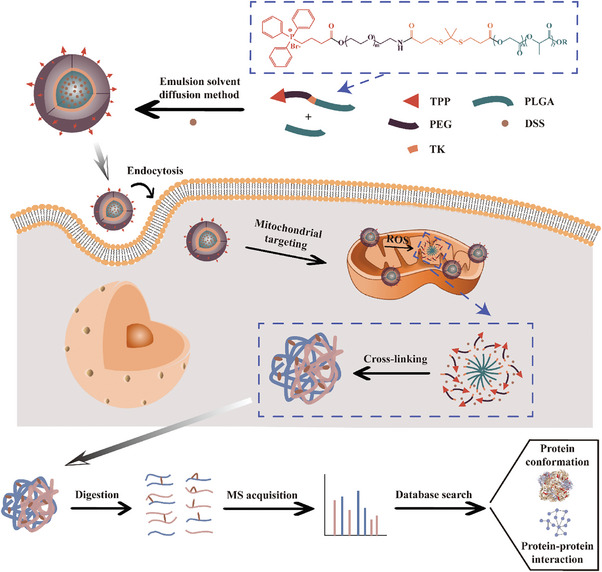
Schematic illustration of R‐CDNP technique and analysis process for native mitochondrial protein conformation and interaction in intact living cells.

In this work, we constructed ROS‐responsive cross‐linker delivery nanoparticles (R‐CDNP) targeting mitochondria by emulsion solvent diffusion method for delivering cross‐linker DSS to mitochondria in living cells. DSS could be triggered to release from R‐CDNP to cross‐link mitochondrial proteins by endogenous ROS. Followed by nano LC‐MS/MS analysis, the analysis of in situ conformations and PPIs of mitochondrial proteins in living HepG2 cells was realized (**Figure** **1**). The cross‐linking distance restraints revealed details about dynamic domain interfaces for chaperone proteins with ATP‐dependent conformation cycles (such as HSPA9), and facilitated the conformation assembly of the prohibitin complex. This study presented the R‐CDNP technique for native mitochondrial protein conformation and interaction analysis in living cells, promising to increase the understanding of mitochondria in complex biological systems.

## 2 Results

### 2.1 Fabrication and Characterization of R‐CDNP

Nanoparticles with mitochondria‐targeting and ROS‐triggered DSS release were prepared by emulsion solvent diffusion method. The key to constructing R‐CDNP is the triphenylphosphine (TPP)‐functionalized block copolymer (TPP‐PEG‐TK‐PLGA) with polyethylene glycol (PEG) block and poly(lactic‐co‐glycolic acid) (PLGA) block using thioketal (TK) bonds as the intermediate linker. The lipophilic cation TPP is used for targeting the inner mitochondrial membrane, and FDA‐approved PLGA and PEG are utilized to load DSS under the premise of biocompatibility, and TK broken in response to endogenous ROS is used to achieve the rapid DSS release in the mitochondria.

TPP‐PEG‐TK‐PLGA was co‐assembled with PLGA to prepare R‐CDNP. The hydrophobic core of polymeric micelles utilizes the hydrophobic interaction to load cargo. Herein, the co‐assembly of PLGA and TPP‐PEG‐TK‐PLGA is to improve the hydrophobicity of whole polymer systems for loading hydrophobic DSS. For DSS with high hydrophobicity, its precipitation rate during the encapsulation was excessively rapid, impeding effective encapsulation. Therefore, the emulsion solvent diffusion method was adopted. The poor solvent ethanol was introduced into the external water phase to make polymer solvent dichloromethane (DCM) diffuse into the external water phase rapidly and induce polymers to accelerate precipitation while delaying DSS precipitation, which further assembled into high DSS‐loaded nanoparticles. At the same time, to prevent the aggregation of nanoparticles, Pluronic F68 was introduced into the internal water phase and the external water phase to maintain stability and positive charge.

Mitochondria‐targeting and ROS‐responsive TPP‐PEG‐TK‐PLGA block polymer was synthesized through a series of sequential conjugation reactions (Figure S1, Supporting Information). The chemical structures and main functional groups of intermediates and the final copolymer TPP‐PEG‐TK‐PLGA were confirmed using NMR and FTIR. ^1^H NMR spectra showed the assignments of characteristic proton peaks (Figures S2 and S3, Supporting Information). The chemical shifts at 8.0–7.6 ppm were ascribed to the protons in the benzene of TPP, and the proton signal of ─CH_2_ in PEG appeared at 3.6 ppm. The peak at 1.5 ppm corresponded to the ─CH_3_ in TK and the ─CH_3_ in PLGA, and two other proton signals of ─CH_2_ and ─CH in PLGA were observed at 4.8 and 5.2 ppm. IR spectra showed the assignments of characteristic absorption bands. As clearly shown in Figure S4 (Supporting Information), the stretching vibration of saturated ─CH in PLGA was observed at 2930 cm^−1^, and the stretching vibration of C═O in the ester bond in PLGA appeared at 1750 cm^−1^. After PEG modification, the stretching vibration of CH_2_ radical group in PEG was observed at 2850 cm^−1^. The stretching vibration of C═C─H and C═C in the benzene of TPP appeared separately at 3050 and 1650–1500 cm^−1^ in the IR spectrum of TPP‐PEG‐TK‐PLGA. Meanwhile, the peak at 995 cm^−1^ was attributed to the P‐C functional group, demonstrating the presence of TPP. And the progress of the reactions was monitored by measuring the weight‐average molecular weight (M_w_) using gel permeation chromatography (GPC). With the sequential bonding of each component (TK, PLGA, PEG, TPP), the shifts of polymer peaks on GPC chromatograms to lower retention time indicated the increase in the molecular weight of the polymers (Figure S5, Supporting Information). Here, the M_w_ of the final TPP‐PEG‐TK‐PLGA copolymer was 12 720 g mol^−1^.

FT‐ICR MS and MALDI‐TOF MS were further used to characterize the structures of polymers. In the negative ion acquisition mode, the [M‐H]^−^ peak of the product TK was found at 251.084 m/z (Figure S6A, Supporting Information), confirming its successful synthesis. For the homopolymer PEG, FT‐ICR MS and MALDI‐TOF MS provided the molecular weight of 1691 and 2065 respectively (Figures S6D and S7A, Supporting Information). But for the copolymer PLGA, only the existence of two repeating units (with masses of 58 and 72) could be observed (Figures S6B and S7B, Supporting Information). This was attributed to the ionization biases resulting from the presence of repeating units with different physicochemical properties in the copolymeric chain, making the MS characterization of polydisperse samples highly challenging.^[^ ^23^ ^]^ For the final copolymer TPP‐PEG‐TK‐PLGA, the MALDI spectrum displayed a normally distributed peak at m/z 2688 with respect to pure PEG homopolymer (Figure S7C, Supporting Information). This signal was attributed to the bonding of PLGA and PEG. All the above results confirmed the successful synthesis of TPP‐PEG‐TK‐PLGA.

The ROS‐responsiveness of TPP‐PEG‐TK‐PLGA copolymers was characterized by GPC to illustrate the M_w_ change of copolymers under ROS stimulation. When exposed to 10 mM H_2_O_2_ for 24 h, GPC analysis showed that TPP‐PEG‐TK‐PLGA was degraded due to the ROS‐responsive cleavage of the TK group (Figure S8, Supporting Information). The ROS level in normal tissues is fairly low (≈20 nm),^[^ ^24^, ^25^ ^]^ where the polymer maintained the integrity after treatment with 20 nM H_2_O_2_ for 24 h, consistent with the GPC profile of TPP‐PEG‐TK‐PLGA prior to the addition of H_2_O_2_ (Figure S8, Supporting Information). It revealed that the general H_2_O_2_ concentration of normal tissues is not enough to trigger the cleavage of TK group.

**Figure 2:**
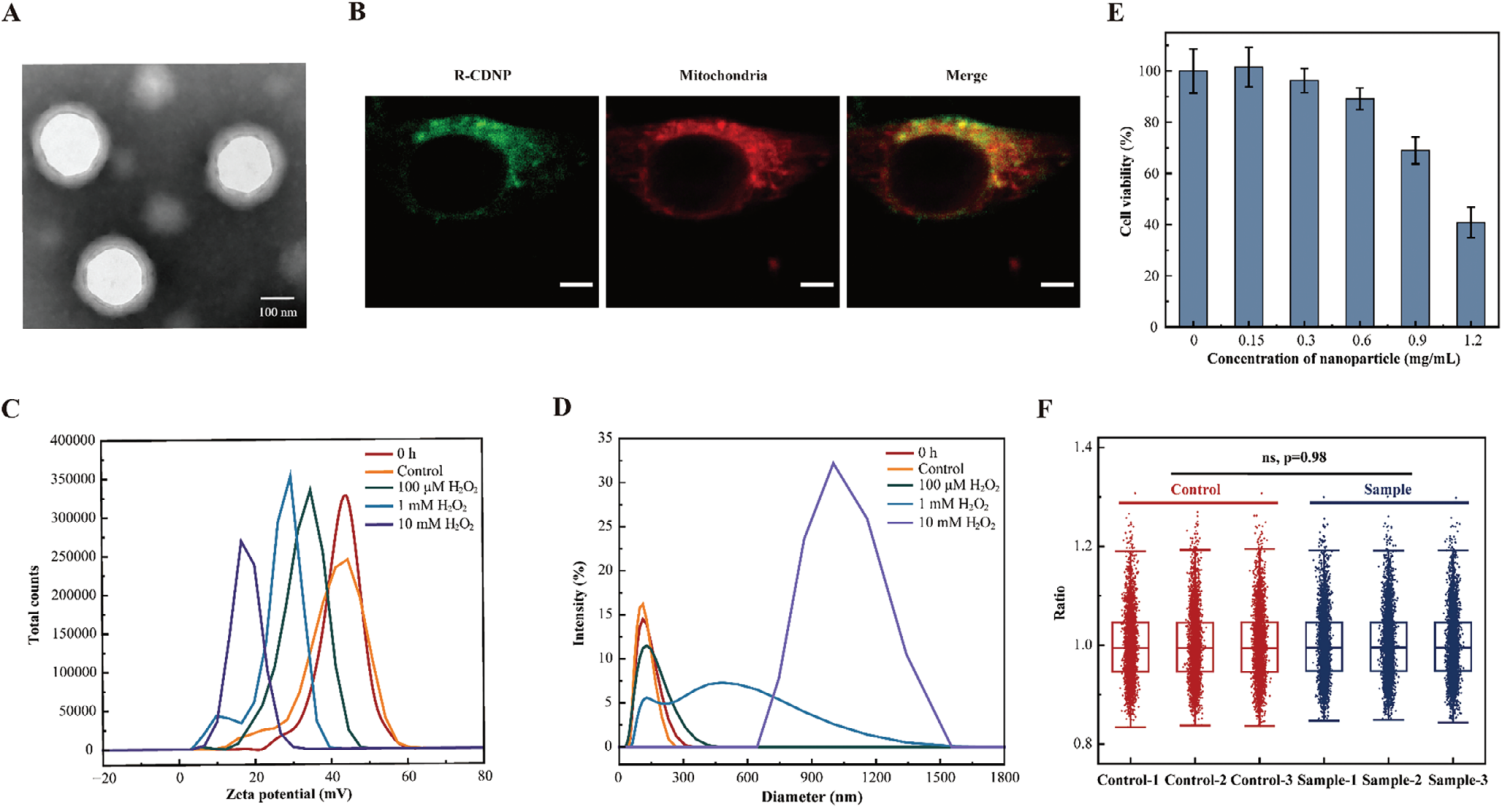
Characterization of R‐CDNP. A) TEM image of R‐CDNP (scale bar = 100 nm). B) CLSM images of HepG2 cells after incubating with Coumarin 6‐labeled R‐CDNP for 3 h. Coumarin 6 (green channel) was utilized to label nanoparticles, and MitoTracker Deep Red (red channel) was employed to co‐stain mitochondria (scale bar = 5 µm). C) Zeta potential changes of R‐CDNP against different H_2_O_2_ concentrations (20 nM, 100 µM, 1 mm, 10 mm) after 7 h. The general H_2_O_2_ concentration (20 nM) of normal tissues was considered as the control group. D) Diameter changes of R‐CDNP against different H_2_O_2_ concentrations (20 nm, 100 µm, 1 mm, 10 mm) after 24 h. The general H_2_O_2_ concentration (20 nM) of normal tissues was considered as the control group. E) Viability of HepG2 cells for varied concentrations of R‐CDNP upon 7 h treatment (mean ± SD, *n* = 4 independent experiments). F) Boxplot showing the proteomic change of HepG2 cells. HepG2 cells were treated with 0.6 mg mL^−1^ R‐CDNP for 7 h, and untreated HepG2 cells were seen as the control group (*n* = 3 independent experiments, two‐tailed Student's *t*‐test).

To obtain the nanoparticles with positive zeta potential, small diameter and high DSS loading efficiency, various preparation parameters were optimized for the formulation of DSS‐loaded nanoparticles, such as polymer composition, water/oil phase composition, and emulsion condition (Table S1, Supporting Information). Transmission electron microscopy (TEM) image showed that the resultant R‐CDNP possessed a uniform spherical sphere with a ring of hydrophilic PEG shells, which could maintain the stability of nanoparticles (**Figure** **2**A). Dynamic light scattering (DLS) analysis revealed that the resultant R‐CDNP prepared under the optimal conditions had a hydrodynamic diameter of 146.6 ± 3.208 nm, a PDI value of 0.187 ± 0.005 and a positive zeta potential of +31.4 ± 0.850 mV, which is beneficial to be uptake by mitochondria.^[^ ^26^ ^]^ The DSS loading efficiency was investigated by high‐performance liquid chromatography (HPLC), and the results showed that the DSS loading efficiency of R‐CDNP was 8.75% (Table S1, Supporting Information).

Confocal laser scanning microscope (CLSM) was employed to visualize the mitochondria‐targeting property of nanoparticles. The fluorescent reagent coumarin 6 was used to label R‐CDNP for intracellular tracking of nanoparticles. HepG2 cells were treated directly with Coumarin 6, which was uniformly distributed within the cells and showed poor colocalization with the mitochondria (a Pearson correlation coefficient of 0.62) (Figure S9A, Supporting Information). Hence, it demonstrated that coumarin 6 did not possess the mitochondria‐targeting ability, precluding any potential confounding effects on assessing the mitochondrial aggregation of R‐CDNP. Upon incubation for 3 h, R‐CDNP and the mitochondria of HepG2 cells exhibited a good overlap (Figure 2B). The Pearson correlation coefficient was 0.88, demonstrating that the TPP moieties of nanoparticles could guide the mitochondria‐targeting property, thereby facilitating the specific accumulation of R‐CDNP at the mitochondrial region. The subcellular distribution of R‐CDNP in intracellular organelles (endoplasmic reticulum, Golgi apparatus, and lysosome) was further characterized to convincingly prove the active mitochondrial targeting of R‐CDNP. When HepG2 cells were treated with Coumarin 6‐labeled R‐CDNP for 3 h, the intracellular distribution of R‐CDNP showed weak colocalization with lysosome (a Pearson correlation coefficient of 0.44), Golgi apparatus (a Pearson correlation coefficient of 0.59), and endoplasmic reticulum (a Pearson correlation coefficient of 0.45) (Figure S9B,C,D, Supporting Information).

To investigate the ROS‐responsiveness, DLS was utilized to monitor the degradation of R‐CDNP. Under ROS stimulation, the zeta potential of nanoparticles decreased sharply, which was attributed to the breakaway of TPP‐PEG due to TK cleavage (Figure S10A, Supporting Information). When treated with 100 µM H_2_O_2_, a comparable ROS level in cancer cells, the zeta potential of nanoparticles decreased from 43.3 to 32.3 mV (Figure 2C), revealing the degradation of R‐CDNP in a simulated microenvironment of cancer cells. The zeta potential of nanoparticles could further decrease to 26.4 and 16.6 mV when H_2_O_2_ concentration rose to 1 and 10 mM respectively, indicating that the degradation degree was in a ROS concentration‐dependent manner. The diameter of R‐CDNP was increased from 126.9 to 245.0, and 1590 nm when exposed to 1 and 10 mM H_2_O_2_ respectively (Figure 2D), showing the significant change of the nanoparticles after the breakage of R‐CDNP. With the increased time of ROS stimulation, the diameter of R‐CDNP continuously increased, which was attributed to the loose spherical structure after the cleavage of the TK linker (Figure S10B, Supporting Information).

In addition, TEM was utilized to directly visualize the morphological change of R‐CDNP. It showed that R‐CDNP was still a uniform spherical structure with a diameter of ≈85 nm in the absence of H_2_O_2_ (Figure S11A, Supporting Information). From the TEM images shown in Figure S11 (Supporting Information), it was observed that R‐CDNP expanded into larger spherical structures after being oxidized at 100 µm and 1 mM H_2_O_2_. Spherical structures were hard to find in the visual field in the presence of 10 mm H_2_O_2_ for 48 h. These supported our conclusion that R‐CDNP could be degraded in the presence of ROS.

The cleavage of R‐CDNP in general H_2_O_2_ concentration (20 nM) of normal tissues was further evaluated. Compared with R‐CDNP prior to the addition of H_2_O_2_, when treated with 20 nM H_2_O_2_ (considered as the control group), the zeta potential change of R‐CDNP was negligible and remained at 38.7 mV (Figure 2C), and the diameter of R‐CDNP remained stable and constant at 117.9 nm (Figure 2D). TEM showed that R‐CDNP maintained a complete spherical structure at 20 nM H_2_O_2_, and the diameter remained relatively stable at 89 nm without morphological changes (Figure S11B, Supporting Information). All the above results confirmed that the TK‐based R‐CDNP was almost incapable of ROS‐triggered response under general H_2_O_2_ concentrations (20 nM) in normal tissues.

Moreover, in vitro DSS release was investigated. Compared with PBS solution, R‐CDNP that treated with 100 µM H_2_O_2_ exhibited the most effective DSS release, with the cumulative DSS release reaching 41.70% within 4 h (Figure S12A, Supporting Information). The simulative cross‐linking of bovine serum albumin (BSA) was further performed to display the ROS‐triggered DSS release. Compared with PBS environment, the weakened BSA monomer bands and the more obvious BSA dimer bonds caused by cross‐linking could be observed when R‐CDNP and BSA were incubated in 100 µm H_2_O_2_ (Figure S12B, Supporting Information), which further demonstrated the release of DSS caused by the cleavage of TK linkages. The combination of the above results indicated that ROS could mediate R‐CDNP to release DSS in a simulated intracellular ROS concentration of 100 µM H_2_O_2_.

CLSM observations revealed that R‐CDNP took 3 h for cellular uptake and mitochondrial targeting. The DSS release curve of R‐CDNP in 100 µ H_2_O_2_ tended to be relatively flat after a 4 h treatment period, suggesting minimal impact from further extending the incubation time on DSS release. Therefore, taking into account both 3 h for mitochondrial localization and 4 h for DSS release triggered by endogenous ROS in the mitochondria, an optimal total incubation time of 7 h for R‐CDNP was determined.

To ensure that mitochondrial protein information was obtained under low disturbance of living cells, the biocompatibility of R‐CDNP was investigated. When incubated with R‐CDNP below the concentration of 0.6 mg mL^−1^ for 7 h, the viability of HepG2 cells was more than 90% (Figure 2E). Label‐free quantitative proteomics further showed that cells treated with 0.6 mg mL^−1^ R‐CDNP for 7 h had a 0.22% of two‐fold significantly different proteins (Figure 2F). All these results indicated the good biocompatibility of R‐CDNP to achieve the in vivo capture of mitochondrial protein information.

### 2.2 ROS‐Triggered Cross‐Linker Release in Mitochondria of Living Cells

**Figure 3:**
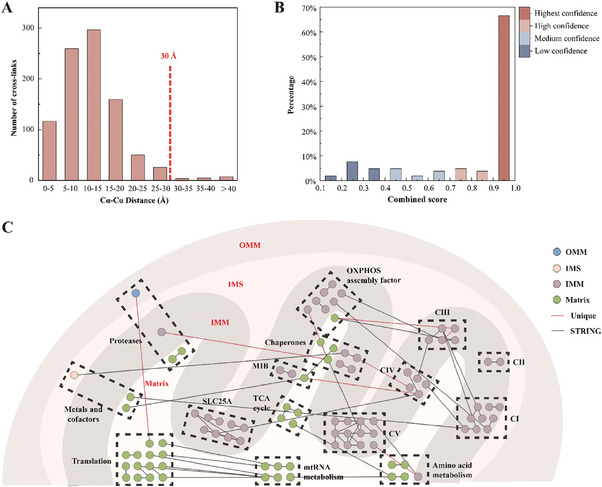
Cross‐linked mitochondrial proteome of HepG2 cells. A) Cα‐Cα distance distribution of identified cross‐links when mapped onto the structures in the PDB database. B) Score distribution of identified PPIs belonging to the STRING database. C) Interaction network between selected groups of mitochondrial proteins. CI = Complex I, CII = Complex II, CIII = Complex III, CIV = Complex IV, CV = Complex V. Nodes were colored based on the submitochondrial localization of proteins, blue for OMM (outer mitochondrial membrane), pink for IMS (intermembrane space), purple for IMM (inner mitochondrial membrane), and green for Matrix. Lines were colored according to interactions found in the STRING database (black) or not (red).

R‐CDNP was added into the cell culture medium and incubated with HepG2 cells for 7 h. After cell lysis and sample pretreatment, the cross‐linked peptides were identified by nanoLC‐MS/MS. 2103 cross‐linked sites of 572 mitochondrial proteins were identified under the identification threshold of 1% FDR and PSM ≥ 2, of which 18.31% (385 sites) were inter‐links between proteins and 81.69% (1718 sites) were intra‐links within the protein (Figure S13, Supporting Information). The cross‐linked proteins covered 32.10% of the constructed mitochondrial proteome database with protein abundance spanning 6 orders of magnitude (Figure S14, Supporting Information),^[^ ^27^ ^]^ achieving the deep coverage of mitochondrial proteins. Gene Ontology biological process (GOBP) analysis revealed that the identified cross‐linked proteins participated in various important pathways, involving OXPHOS, mitochondrial translation, mitochondrial organization, TCA cycle, fatty acid beta‐oxidation, et al (Figure S15A, Supporting Information). 926 cross‐links could be mapped on the protein crystal structures of PDB database, and the remaining could not be mapped due to the lack of crystal structures in PDB database. The Cα‐Cα solvent accessible surface distances were counted, and the proportion of cross‐links within the maximum distance restraint of DSS (30 Å) was 98.27% (**Figure** **3**A). Such a high degree of matching demonstrated that the obtained cross‐links were highly credible and could provide reliable information for protein conformation analysis.

Additionally, 385 inter‐links corresponded to 142 PPIs among 181 mitochondrial proteins, and 105 PPIs were reported in the STRING database, among which 70 PPIs were of the highest confidence with a combined score over 0.9 (Figure 3B), which indicated that this technique could offer reliable PPIs. As for the 37 pairs of interacting proteins not reported in the STRING database, their similarities of cellular component (CC), molecular function (MF), and biological process (BP) were calculated. 27 pairs showed correlation (similarities ≥ 0.4), which suggested that they had potential interactions (Table S2, Supporting Information). GOBP analysis revealed that the interacting proteins not reported in the STRING database were mainly involved in mitochondrion organization, hydrogen ion and calcium ion transmembrane transport, regulation of cell communication, calcium channel activity, and protein refolding (Figure S15B, Supporting Information). Hence, these previously unrevealed interacting proteins probably belonged to weak or transient PPIs, which could be captured in an unperturbed microenvironment of living cells. Identified inter‐links covered all compartments of mitochondria organization. Examples of mitochondrial proteins with different submitochondrial localizations were displayed in the interaction network, including IMM‐spanning OXPHOS complexes, membrane‐localized SLC25A carrier family, OMM‐IMM‐spanning mitochondrial intermembrane space bridging complexes (MIB), mainly matrix‐localized TCA cycle proteins and mitoribosome, and scattered chaperones (Figure 3C). The cross‐linking information of these proteins was identified (Figures S16–S19, Supporting Information), and the majority of identified intra‐links could be consistent with protein structures of PDB database and AlphaFold structures, showing their good compatibility with these structures (Figures S16B, S17B, S18B, and S19, Supporting Information). Beyond that, the C‐terminal domain of MIB protein subunit IMMT, a crucial region for the formation of crista junctions,^[^ ^28^ ^]^ was identified to interact with MICOS10 (Figure S17A, Supporting Information), which may jointly affect the formation of crista junctions and cristae morphology.

### 2.3 Protein Conformation and Interaction Analysis of OXPHOS Complexes

**Figure 4:**
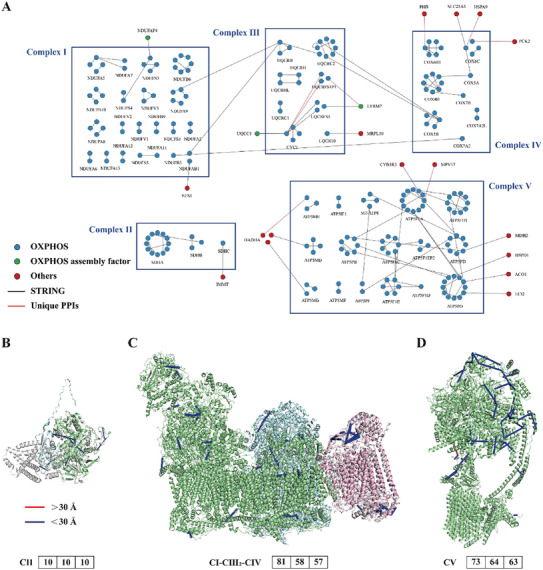
The cross‐linking information among OXPHOS complexes. A) The interaction network. These nodes were indicated using different colors, blue for OXPHOS subunits, green for OXPHOS assembly factors, and red for others. The interactions reported in the STRING database were shown in black, and unreported interactions (unique PPIs) were in red. B) Mapping of identified cross‐links onto CII. Human SDHA structure (PDB: 6VAX shown in palegreen) and SDHB structure (AlphaFold shown in palecyan) were positioned using porcine complex II (PDB: 4YXD shown in gray) as a template. C) Mapping of identified cross‐links onto CI‐CIII2‐CIV. The bovine CIV of CI‐CIII2‐CIV (PDB: 5XTH. CI, CIII, and CIV were in palegreen, palecyan, and gray) was replaced with human CIV (PDB: 5Z62 shown in lightpink). D) Mapping of identified cross‐links onto bovine CV (PDB: 6ZQM shown in palegreen). The numbers in the box from left to right represented the number of identified cross‐links, the number of cross‐links mapped onto structures, and the number of cross‐links satisfying the distance restraint. Blue lines represented the cross‐links satisfying the distance restraint, and red lines represented the cross‐links exceeding the distance restraint.

OXPHOS is an important metabolic pathway, and the released energy is used to generate ATP, which is a hallmark of mitochondria.^[^ ^3^ ^]^ 57 protein subunits of IMM‐spanning OXPHOS complexes were shown (**Figure** **4**A). The inter‐link between NDUFB3 (the subunit of Complex I) and COX7A2 (the subunit of Complex IV) was identified, providing experimental evidence to the data in the STRING database obtained by co‐expression and textmining.^[^ ^29^ ^]^ Besides, the inter‐links also provided the experimental evidence for UQCRHL and UQCRH, CYC1 and UQCC3, UQCR10 and MRPL30, ATP5PO and ECI2.

Complex II is an enzyme which links the TCA cycle and OXPHOS.^[^ ^30^, ^31^ ^]^ The crystal structure of Complex II from pigs (PDB: 4YXD) was used as a template, and then the crystal structure of human SDHA (PDB: 6VAX) and AlphaFold structure of SDHB were positioned. 10 cross‐links could successfully be mapped to the constructed structure, and all satisfied the DSS distance restraint (Figure 4B).

Complex I, Complex III and Complex IV are assembled into human respiratory supercomplex I1III2IV1. The bovine‐derived crystal structure of Complex IV in I1III2IV1 (PDB: 5XTH) was replaced by human Complex IV (PDB: 5Z62). 58 cross‐links that were mapped to the reconstructed crystal structure showed excellent compatibility (Figure 4C). Self‐cross‐linking at the same site K159 of UQCRC2 (the subunit of Complex III) was identified with a Cα‐Cα distance of 21.03 Å, indicating that Complex III formed a multimer, consistent with the result reported in previous literature.^[^ ^13^ ^]^ 23 cross‐links couldn't be mapped to the reconstructed structure. Of the 12 intra‐links, 8 and 2 intra‐links were caused by the lack of N‐terminus and C‐terminus of protein subunits in the reconstructed crystal structure respectively, and 2 intra‐links were caused by the complete absence of protein subunits. Then all of them were mapped to AlphaFold structures, and 11 intra‐links were found to satisfy DSS distance restraint (Figure S20A, Supporting Information), demonstrating that our technique could provide protein conformation information of N‐terminus and C‐terminus. For 11 inter‐links, 4 inter‐links could not be resolved due to the complete absence of protein subunits, and the remaining 7 inter‐links revealed 6 pairs of experimentally determined PPIs in the STRING database. These 7 inter‐links were further explored by aligning AlphaFold structures to the residues of the reconstructed crystal structure (Figure S20B, Supporting Information), of which 3 inter‐links that unsatisfied DSS distance restraint were K4 of UQCRB and K92 of UQCRC2 (35.1 Å), K80 of NDUFA7 and K46 of NDUFA5 (32.1 Å), and K2 of COX6C and K72 of COX5A (38.3 Å). The first cross‐linked sites were also identified by the method previously developed by our research group.^[^ ^16^ ^]^ These cross‐links with distance conflict may be due to the flexibility of conformation.

Complex V, ATP synthase, drives the phosphorylation of ADP to ATP. We mapped cross‐links to the bovine‐derived crystal structure of Complex V (PDB: 6ZQM) in the absence of human structure. Identified cross‐links could match well with the crystal structure (Figure 4D). 6 unmatched intra‐links were mapped to AlphaFold structures with a good matching degree (Figure S20C, Supporting Information). 2 unmatched inter‐links (K54 of MT‐ATP8 and K105 of ATP5PF, and K49 of MT‐ATP8 and K25 of ATP5PD) corresponded to two experimentally determined PPIs of MT‐ATP8 in the STRING database. The missing parts of the crystal structure were filled in by AlphaFold structures, and 2 inter‐links beyond the distance restraint were linked to the C terminus of MT‐ATP8 (Figure S20D, Supporting Information). The structure of MT‐ATP8 is predicted to contain a flexible and disordered C‐terminal tail.^[^ ^13^ ^]^ Based on this prediction, it may be in dynamic contact with the surfaces of ATP5PF and ATP5PD and thus be cross‐linked, indicating that our technique could capture dynamic interactions. In short, our technique not only provided experimental evidence of interactions and complementary information on complex conformation, but also captured dynamic conformations and interactions.

### 2.4 Dynamic Conformation Analysis of Chaperones

Chaperones, dynamic molecular machines, contribute to the proper folding and assembly of mitochondrial proteins, and maintain mitochondrial homeostasis. It is reported that prohibitin 1 (PHB1) and prohibitin 2 (PHB2) assembled into ring‐shaped prohibitin complexes in the IMM, which have connections with the activity of OXPHOS.^[^ ^32^ ^]^ The identification of 8 inter‐links between PHB1 and PHB2 further confirmed the formation of prohibitin complex, and cross‐linked sites provided the distance constraints information for the structural assembly of the complex. The cross‐link of PHB1 and COX6B1 (a subunit of Complex IV) was identified, and the cross‐link of PHB2 and CYCS (the electron carrier protein) was identified (Figure S21, Supporting Information). It was further suggested that there was a close relationship between prohibitins and mitochondrial respiration.

**Figure 5:**
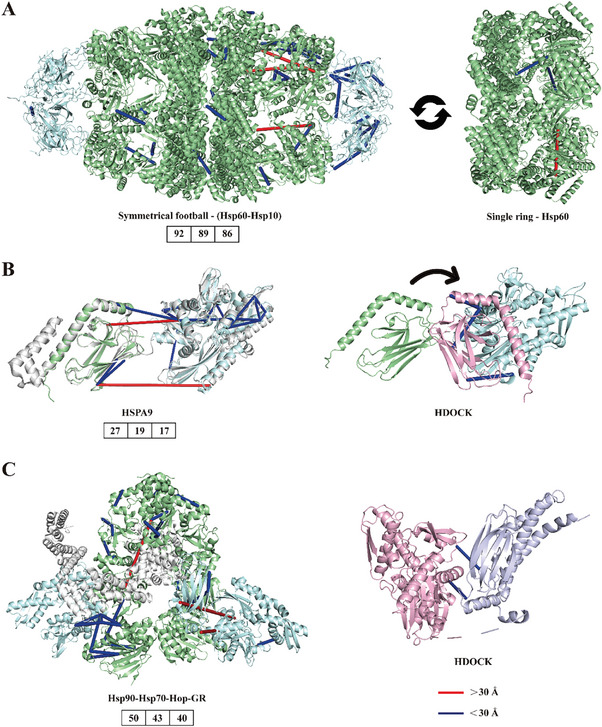
The cross‐linking information for chaperonin proteins with ATP‐dependent conformation cycles. A) Mitochondrial chaperonin complex. Cross‐links were mapped on the symmetrical football complex (PDB: 4PJ1, left structure) containing HSP60 (shown in palegreen) and HSP10 (shown in palecyan). Cross‐links exceeding distance restraint were mapped on the single ring HSP60 complex (PDB: 7AZP shown in palegreen, right structure). B) Human stress‐70 protein. E.coli HSP70 structure (PDB: 2KHO shown in gray, left structure) was used as a template while positioning SBD (PDB: 3N8E shown in palegreen) and NBD (PDB: 6P2U shown in palecyan) of human stress‐70 protein. Cross‐links were mapped on the constructed protein structure. HDOCK was used to dock SBD and NBD domains by the distance restraints at the domain interface, yielding a domain arrangement (shown in lightpink, right structure). Cross‐links at the domain interface were mapped on HDOCK structure. C) Heat shock 70 kDa protein 1A. Cross‐links were mapped on the inactive “client‐loading” complex (PDB:7KW7, left structure) containing HSP70 (shown in palecyan) and HSP90 (shown in palegreen). HDOCK was used to dock SBD (PDB: 4PO2 shown in bluewhite) and NBD (PDB: 7Q4R shown in lightpink) domains of HSP70 by the distance restraints at the domain interface. Cross‐links at the domain interface were mapped on HDOCK structure. Blue lines represented the cross‐links satisfying the distance restraint, and red lines represented the cross‐links exceeding the distance restraint.

60 kDa heat shock protein (HSP60) and its cochaperonin 10 kDa heat shock protein (HSP10) construct the human mitochondrial chaperonin complex. The apo form of HSP60 as a single‐ring complex and football‐shaped double‐ring complex containing HSP60 and HSP10 are two main forms in the nucleotide‐dependent chaperonin folding reaction cycle.^[^ ^33^, ^34^ ^]^ Cross‐links were mapped onto the structure of a symmetric football‐shaped intermediate (PDB: 4PJ1) (**Figure** **5**A, left structure), and 3 cross‐links beyond DSS distance restraint were all in the HSP60 ring. By mapping these 3 cross‐links onto the structure of apo form HSP60 (PDB:7AZP), 2 distance conflicts were resolved, and the Cα‐Cα distance of the other was significantly shortened (Figure 5A, right structure). It may be that the subunits of mitochondrial chaperonin ring have unconcerted movements when binding or dissociating with substrate proteins and the apical domains of HSP60 have high flexibility, which is similar to the results reported in previous literature.^[^ ^15^, ^33^, ^35^ ^]^

70 kDa heat shock protein (HSP70) with the N‐terminal nucleotide‐binding domain (NBD) and C‐terminal substrate‐binding domain (SBD) has ATP‐dependent conformational cycles.^[^ ^36^, ^37^ ^]^ Human stress‐70 protein (HSPA9) is a member of HSP70 family. The structures of human NBD (PDB: 6P2U) and SBD (PDB: 3N8E) of HSPA9 were positioned to the structure of Escherichia coli in the ADP binding state (PDB: 2KHO). Identified cross‐links were mapped to the above‐constructed structure (Figure 5B, left structure), where 2 cross‐links exceeding DSS distance restraint were at the interface of two domains. HDOCK was used to dock these two domains according to cross‐linking restraints, which yielded a domain arrangement. It was found that cross‐links at the interface of two domains could meet DSS distance restraint (Figure 5B, right structure). In addition, heat shock 70 kDa protein 1A (HSPA1A) also belongs to HSP70 family, which together with 90 kDa heat shock protein (HSP90) promotes the folding and maturation of client proteins. Identified cross‐links were mapped onto the inactive “client loading” complex Hsp90‐Hsp70‐Hop‐GR, and most of cross‐links met DSS distance constraints (Figure 5C, left structure). 2 of 3 long‐distance cross‐links were interdomain cross‐links between NBD and SBD of HSP70, and the remaining one was inside HSP90. Due to the lack of an independent crystal structure of HSP70, we used HDOCK to dock NBD and SBD of HSP70 according to the distance constraints of interdomain cross‐links. Then the distance conflicts of two interdomain cross‐links were resolved (Figure 5C, right structure), indicating that the distance conflicts may be caused by the conformational change and docking of these two domains in the ATP‐dependent conformational cycles. Therefore, this technique contributes to elucidating the molecular mechanism of substrate binding and allosteric coupling.

## 3 Discussion

Mitochondria have a complex proteome, and more than 150 distinct diseases are associated with mitochondrial protein dysfunction.^[^ ^38^ ^]^ Protein function is largely determined by 3D structures and formed PPIs.^[^ ^7^ ^]^ Clarifying mitochondrial protein structures and PPIs can help reveal the mechanism of mitochondrial dysfunction. In this study, we constructed R‐CDNP to achieve ROS‐triggered cross‐linker release in mitochondria in response to endogenous ROS. Without genetically modifying cells or isolating mitochondria, R‐CDNP captured in situ mitochondria information in intact living cells without perturbation, thus providing information on mitochondrial protein conformation and interaction in a closely native microenvironment.

Mitochondria‐targeting and ROS‐responsive polymer is the key to constructing R‐CDNP. Mitochondria‐targeting group TPP and ROS‐responsive group TK were conjugated with biocompatible PLGA and PEG to construct TPP‐PEG‐TK‐PLGA polymer. It is well‐known that the hydrophobic core of polymeric micelles utilizes the hydrophobic interaction to carry out hydrophobic cargos. Herein, TPP‐PEG‐TK‐PLGA was co‐assembled with PLGA to enhance the hydrophobicity of whole polymer systems to facilitate DSS loading. To reduce the negative effects on ROS‐responsiveness and positive zeta potential of constructed nanoparticles, PLGA was incorporated with a small proportion of 20%. For emulsion solvent evaporation method, the precipitation rate of hydrophobic DSS was much faster than the formation rate of nanoparticles, hindering the effective DSS encapsulation. Consequently, emulsion solvent diffusion method, a combination of emulsion solvent evaporation method and nanoprecipitation method, was utilized to achieve the high DSS loading efficiency. The poor solvent ethanol was introduced into the external water phase to expedite polymer precipitation and delay DSS precipitation. However, the nanoparticles would aggregate into large particles when the polymer precipitation occurred too rapidly. Therefore, surfactants were added into the internal water phase and the external water phase to increase the solution viscosity and maintain the nanoparticle stability. Biocompatible surfactant F68 was ultimately chosen to construct R‐CDNP without affecting the positive charge (Table S1, Supporting Information). These aforementioned characteristics guaranteed the successful development of R‐CDNP, which supported the cross‐linker release in the mitochondria of living cells.

When R‐CDNP were treated with 100 µM H_2_O_2_, a comparable ROS level in cancer cells, the decrease of zeta potential from 43.3 to 32.3 mV and the cumulative DSS release of ≈41.70% for 4 h demonstrated the ROS‐responsiveness of R‐CDNP. CLSM showed that R‐CDNP and mitochondria exhibited good co‐localization with a Pearson correlation coefficient of 0.88, confirming the mitochondria‐targeting property of R‐CDNP. CCK8 assay and label‐free quantitative proteomics indicated that R‐CDNP were biocompatible, and 0.6 mg mL^−1^ R‐CDNP with 7 h incubation was the ideal safety limit. After cellular internalization, R‐CDNP could actively anchor to IMM after lysosomal escape, where they were triggered to release DSS via the cleavage of TK group under mitochondrial ROS stimulation. Then DSS cross‐linked mitochondrial proteins in an undisturbed and biocompatible way.

In total, we identified 2103 cross‐linked sites of 572 mitochondrial proteins under the identification threshold of 1% FDR and PSM≥2, of which 18.31% (385 sites) were inter‐links and 81.69% (1718 sites) were intra‐links. Identified proteins covered all compartments of mitochondria, and most mitochondrial proteins were localized in the mitochondrial matrix and IMM (Figure 3C), which may indicate that R‐CDNP anchored IMM and released DSS for cross‐linking reaction under the stimulation of mitochondrial ROS.

Compared with the previous study of our research group (CDNP),^[^ ^16^ ^]^ R‐CDNP has four main advantages. First, the R‐CDNP exhibited a small average diameter of 146.6 nm compared to 275 nm for CDNP, which is more conducive to mitochondrial uptake. Second, R‐CDNP had a faster DSS release rate at the mitochondria. R‐CDNP exhibited a trend of rapid release with a cumulative DSS release of 41.70% within 4 h in the presence of 100 µM H_2_O_2_, while CDNP in the previous study only released 20.40%. Furthermore, the time period that was required to release 50% of encapsulated DSS (t50) in the Duvvuri kinetic model was calculated. The t50 of R‐CDNP was 9.619 h, while the t50 of CDNP was 11.235 h. Third, due to the enhanced DSS release rate, R‐CDNP allowed for the decrease of nanoparticle concentration down to 0.6 mg mL^−1^ (compared to 1.8 mg mL^−1^ of CDNP), which confer an advantage on biocompatibility. Lastly, compared with CDNP (1203 cross‐linked peptides under 1% FDR), R‐CDNP captured 2103 cross‐linked sites of 572 mitochondrial proteins under the identification threshold of 1% FDR and PSM≥2, showing more abundant cross‐linking information. This may be due to the higher DSS release rate of R‐CDNP in mitochondria, leading to better cross‐linking coverage.

98.27% of identified cross‐links that were mapped to the crystal structures in the PDB database were within the DSS distance constraint, providing reliable protein conformation information. Cross‐links were evenly distributed in protein conformations and had good coverage for both N‐terminus and C‐terminus of proteins, which could provide complementary information for other structural elucidation methods. The distance restraints offered by the inter‐links of PHB1 and PHB2 could be conductive to the docking assembly of PHB complex, suggesting the value in complex conformations that lack experimental characterization. Experimental results on chaperone proteins with ATP‐dependent conformational cycles indicated that chaperone proteins were highly flexible and underwent open‐closed conformational transitions during substrate binding and dissociation.

The combined scores of 66.67% of identified PPIs belonging to the STRING database exceeded 0.9, showing the high credibility of identified PPIs. The experimental results of Complex V showed that two unmatched inter‐links both involved the C‐terminus of MT‐ATP8, which may be caused by the fact that the C‐terminal tail was in dynamic contact with ATP5PF and ATP5PD. These data demonstrated the ability to capture dynamic interactions and provide detailed interaction sites. In addition, 37 pairs of interacting proteins that were not reported in the STRING database were also identified, among which 27 pairs of interacting proteins had similarities of ≥ 0.4 on CC, MF, and BP, and were considered to have potential interactions. For example, identified ATAD3C and ATAD3A are family members of ATPase proteins associated with various cellular activities, and ATAD3C is incorporated into ATAD3A complex to complete mitochondrial membrane organization.^[^ ^39^ ^]^ Also identified ATP5F1C and ATP5F1EP2 are subunits of mitochondrial membrane ATP synthetase and participate in ATP synthesis. Therefore, these two pairs of interacting proteins that have not been reported in the STRING database may interact to a large extent.

To improve the controllability of cross‐linker release and capture more abundant cross‐linking information, this technology needs further development. On the one hand, multiple stimuli‐responsive nanoparticles will be investigated to improve response sensitivity and promote cross‐linker release, thus enhancing the cross‐linking coverage. On the other hand, universal cross‐linker nanoparticles will be developed to load various cross‐linkers with different reaction groups and enrichable tags, thereby achieving the complementarity of cross‐linking information to increase the accuracy and depth of cross‐linking.

Briefly, we developed R‐CDNP for mitochondria‐specific cross‐linker release, enabling the analysis of native mitochondrial protein conformations and interactions in intact living cells. This technique could be hopeful for extending the study of the mitochondrial proteome to tissues or living organisms.

## 4 Experimental Section

### Synthesis of TPP‐PEG‐TK‐PLGA Polymer

ROS‐responsive TK linker was synthesized according to the following route. 3‐mercaptopropionic acid (47.2 mmol) (Acros Organics, Thermo Fisher Scientific) and a catalytic amount of trifluoroacetic acid (20 µL) (Sigma‐Aldrich, USA) were dissolved in acetone (21.46 mmol) (Kemiou, Tianjin, China) and stirred at room temperature for 8 h. The mixture was placed on ice for 1 h until the crystallization was completed. Then white precipitation was washed with hexane and cold water, and the product was lyophilized. ^1^H NMR, FTIR, and MS characterizations were performed. ESI‐MS: *m/z* calc. for C_9_H_16_O_4_S_2_ 252.049; found 251.084 [M‐H]^−^.

PLGA‐TK was synthesized according to the following route. Under nitrogen protection, TK (0.5 mmol), 4‐Dimethylaminopyridine (DMAP, 1 mmol) (Aladdin, Shanghai, China) and ester‐terminated poly(lactic‐co‐glycolic acid) (PLGA, M_w_ = 7000, LA: GA = 50:50, 0.05 mmol) (Daigang, Shandong, China) were dissolved in anhydrous dichloromethane (DCM, 7 mL). N, N’‐Dicyclohexylcarbodiimide (DCC, 1 mmol) (Sigma‐Aldrich, St Louis, USA) was dissolved in anhydrous DCM (3 mL) and added to the above mixed solution slowly at 0 °C, stirring at room temperature for 48 h. Subsequently, insoluble dicyclohexylurea was filtered by 0.22 µm filter membranes. The solvent was removed by rotary evaporation and redissolved in anhydrous DCM (2 mL). The concentrated solution was dropped into cold methanol to precipitate the product. The precipitate was washed with cold methanol three times and vacuum‐dried. ^1^H NMR, FTIR, and MS characterizations were performed. The M_w_ of PLGA‐TK was determined by GPC as 3922 g mol^−1^ relative to polymethyl methacrylate (PMMA).

PLGA‐TK‐PEG was synthesized according to the following route. PLGA‐TK (0.1 mmol), N‐(3‐Dimethylaminopropyl)‐N'‐ethylcarbodiimide hydrochloride (EDC, 0.15 mmol) (J&K, Beijing, China), N‐Hydroxysuccinimide (NHS, 0.15 mmol) (J&K, Beijing, China) and amine polyethylene glycol hydroxyl (H_2_N‐PEG‐OH, M_w_ = 2000, 0.1 mmol) (Pengsheng, Shanghai, China) were dissolved in anhydrous DCM (10 mL) under nitrogen atmosphere. After being stirred at room temperature for 48 h, the solvent was removed by rotary evaporation and anhydrous DCM (2 mL) was added to redissolve the product. Then the solution was dropped into cold 50:50 diethyl ether and methanol to precipitate the product. The product was washed with cold 50:50 diethyl ether and methanol three times and vacuum‐dried. ^1^H NMR, FTIR, and MS characterizations were performed. The M_w_ of PLGA‐TK‐PEG was determined by GPC as 11 606 g mol^−1^ relative to PMMA.

TPP‐PEG‐TK‐PLGA was synthesized according to the following route. PLGA‐TK‐PEG (0.05 mmol), (3‐Carboxypropyl)triphenylphosphonium bromide (TPP, 0.5 mmol) (J&K, Beijing, China) and DMAP (0.5 mmol) were dissolved in anhydrous DCM (7 mL). DCC (0.5 mmol) was dissolved in anhydrous DCM (3 mL) and added to the mixed solution slowly at 0 °C, stirring at room temperature for 48 h. Subsequently, insoluble dicyclohexylurea was filtered by 0.22 µm filter membranes. The solvent was removed by rotary evaporation and redissolved in anhydrous DCM (2 mL). The concentrated solution was dropped into cold diethyl ether to precipitate the product. The precipitate was washed with cold diethyl ether three times and vacuum‐dried. ^1^H NMR, ^31^P NMR, FTIR, and MS characterizations were performed. The M_w_ of TPP‐PEG‐TK‐PLGA was determined by GPC as 12 720 g mol^−1^ relative to PMMA.

### ROS‐Responsive of TPP‐PEG‐TK‐PLGA

The cleavability of thioketal groups in TPP‐PEG‐TK‐PLGA copolymer was evaluated by GPC through the M_w_ change. Briefly, TPP‐PEG‐TK‐PLGA was separately dissolved in ACN containing 20 nM H_2_O_2_ and 10 mM H_2_O_2_ for 24 h, and then the M_w_ of polymers was measured by GPC.

### Preparation of R‐CDNP

An optimization study of appropriate encapsulation method was investigated to achieve efficient disuccinimidyl suberate (DSS) loading. Emulsion solvent evaporation method involved successive adjustments in the mass ratio of polymer to DSS, ultrasonic power and time, polymer concentration, polymer ratio, and the ratio of the oil phase. Additionally, emulsion solvent diffusion method has also been optimized with modifications made to the types of internal water phase, the types and ratio of external water phase, as well as the types of surfactants. Emulsion solvent diffusion method had a higher DSS loading efficiency than emulsion solvent evaporation method (Table S1, Supporting Information). Finally, the modified emulsion solvent diffusion method with optimal parameters was adopted.

Typically, TPP‐PEG‐TK‐PLGA (16 mg), PLGA (4 mg, mass ratio of polymer = 4:1) and (4 mg DSS, mass ratio of polymer: cross‐linker = 5:1) were dissolved in anhydrous DCM (0.4 mL) to a final polymer concentration of 50 mg mL^−1^, and the mixture was added into 1% (w/v) Pluronic F68 solution (4 mL) (Macklin, Shanghai, China) while emulsifying for 2 min by 120 W sonication (4 min, 5 s on, 5 s off) in an ice bath. Formed nanoemulsion was then added into ethanol: 0.4% (w/v) F68 mixed solution (20 mL) (volume ratio = 3:2) and stirred at room temperature for 20 min. The organic solvent was removed by vacuum evaporation at 30 °C, and the solution was centrifuged at 1000 g to remove large particles. The resulting nanoparticles (R‐CDNP) were collected by centrifugation at 40000 g and washed twice with ultrapure water. Coumarin 6‐labeled R‐CDNP were synthesized according to a similar procedure. Briefly, TPP‐PEG‐TK‐PLGA (16 mg), PLGA (4 mg), and Coumarin 6 (0.3 mg) (Sigma‐Aldrich, St Louis, USA) were dissolved in anhydrous DCM (0.4 mL). Subsequent procedures were mentioned above.

### Characterization of R‐CDNP

The hydrodynamic size, size distribution and zeta potential of R‐CDNP were determined by dynamic light scattering (DLS) using the Malvern Zetasizer Nano ZS‐90 instrument (UK). For DLS measurement, all samples suspended in H_2_O were carried out in triplicate at 25 °C. The morphology of R‐CDNP was examined by transmission electron microscopy (TEM) using JEM‐2000EX (Japan) operated at an accelerating voltage of 120 kV. For TEM image, sample solution was applied to a copper grid and stained by 1% (w/v) phosphotungstic acid aqueous solution to finish the sample preparation.

To determine DSS loading efficiency of R‐CDNP, high‐performance liquid chromatography (HPLC) with homemade C18 reversed‐phase column (5 µm, 150 Å, 4.6 mm i.d. × 15 cm) was used. The DSS absorption curve at 200 nm was quantified and the standard calibration curve of DSS was established. Lyophilized nanoparticles were dissolved in DMSO and further evaluated by HPLC analysis based on the standard curve. DSS loading efficiency was calculated as Equation (1):

(1) $$\begin{array}{ccc} \mathrm{Loading}\;\mathrm{efficiency}\;(\%) & = & \left(\mathrm{mass}\;\mathrm{of}\;\mathrm{loaded}\;\mathrm{DSS}\;/\;\mathrm{mass}\;\mathrm{of}\;R-\mathrm{CDNP}\right)\\ & & \times \;100\% \end{array}$$

### Cell Lines and Culture

Human hepatocellular carcinomas (HepG2) were acquired from the cell bank of the Committee on Type Culture Collection of the Chinese Academy of Sciences (CTCC, Shanghai, China). HepG2 cells were cultured in Dulbecco's Modified Eagle's Medium (DMEM) (Gibco, USA) supplemented with 10% (v/v) fetal bovine serum and penicillin (100 U mL^−1^) / streptomycin (100 µg mL^−1^) at 37 °C and 5% CO_2_.

### Mitochondria‐Targeting Property of R‐CDNP

Confocal laser scanning microscope (CLSM) imaging was applied to observe the intracellular distributions of R‐CDNP. Briefly, HepG2 cells were treated with fresh DMEM medium containing Coumarin 6‐labeled R‐CDNP at 37 °C and 5% CO_2_. At a predetermined time interval (3 h), cells were respectively stained with MitoTracker Deep Red FM (0.2 µm) (Invitrogen, Thermo Fisher Scientific) for 30 min, ER‐Tracker Red (500 nM) (Invitrogen, Thermo Fisher Scientific) for 15 min, Golgi‐Tracker Red (10 µM) (Invitrogen, Thermo Fisher Scientific) for 30 min, LysoTracker Red DND‐99 (500 nM) (Invitrogen, Thermo Fisher Scientific) for 30 min. For the control experiment of Coumarin 6, the HepG2 cells were directly incubated with Coumarin 6 for 3 h, and then stained with MitoTracker Deep Red FM (0.2 µM) (Invitrogen, Thermo Fisher Scientific) for 30 min. After being washed thrice with PBS, the cells were imaged by the Andor live cell confocal imaging platform. The channels of Coumarin 6, MitoTracker Deep Red FM, ER‐Tracker Red, Golgi‐Tracker Red, and LysoTracker Red DND‐99 were separately recorded at the excitation wavelength of 488, 640, 561, 561, and 561 nm.

### ROS‐Responsiveness of R‐CDNP

The cleavability of thioketal groups in R‐CDNP was evaluated by the change in zeta potential, diameter and morphology. Briefly, R‐CDNP were incubated against different H_2_O_2_ concentrations (10 mm, 1 mM, 100 µM, 20 nM), and the change of zeta potential and diameter was continuously observed by DLS. In addition, R‐CDNP were incubated against PBS environment and different H_2_O_2_ concentrations (10 mM, 1 mM, 100 µM, 20 nM) for 48 h, and the morphological change was recorded by TEM. 20 nM H_2_O_2_, a general H_2_O_2_ concentration in normal tissues, was considered as the control group.

Then in vitro DSS release was further investigated. R‐CDNP were dispersed in aqueous solutions with or without 100 µm H_2_O_2_ and incubated at 37 °C. The release mediums were centrifugally collected with the 100 kDa ultrafiltration tubes every half hour, and then R‐CDNP were redispersed with an equal volume of fresh mediums. The DSS content in the release mediums was measured by HPLC analysis, and the percentages of cumulative released DSS were plotted against time. Moreover, in vitro cross‐linking behavior of R‐CDNP with BSA was also evaluated upon H_2_O_2_ or PBS environment using SDS‐PAGE gel. The solutions of BSA in 100 µM H_2_O_2_ or PBS were used to disperse R‐CDNP, respectively. Then the mixtures were incubated at 37 °C and constantly taken out at predetermined time intervals (1, 2, 3, and 4 h), which were terminated with ammonium bicarbonate (a final concentration of 50 mM). The sample solutions were mixed with 6 × protein loading buffer (volume ratio = 5:1) and boiled at 95 °C for 5 min. All samples were loaded into the SDS‐PAGE gel (12.5% gel) with a fixed protein amount (4 µg), and the gel was stained with Coomassie blue and recorded by ChemiDoc XPS+ (Bio‐Rad, USA).

### Biocompatibility of R‐CDNP

In vitro cytotoxicity was assessed by CCK8 assays. HepG2 cells were seeded in a 96‐well plate for 24 h. After being replaced with fresh medium, cells were treated with varied concentrations of R‐CDNP (0.15, 0.3, 0.6, 0.9, 1.2 mg mL^−1^) for 7 h. Then CCK8 solution (10%, v/v) (Beyotime, Shanghai, China) was added to each well and incubated for 3 h. The absorbance at 450 nm was measured by a microplate reader. The cell viability was calculated as Equation (2):

(2) $$\begin{array}{c} \mathrm{Viability}\;\left(\%\right)=\left({\mathrm{OD}}_{\mathrm{sample}}-{\mathrm{OD}}_{\mathrm{blank}1}\right)\;/\;\left({\mathrm{OD}}_{\mathrm{control}}-{\mathrm{OD}}_{\mathrm{blank}2}\right)\;\times \;100\% \end{array}$$

where OD_sample_ is obtained in the presence of R‐CDNP with cells, OD_blank1_ is obtained in the presence of R‐CDNP with DMEM medium, OD_control_ is obtained in the presence of pure cells, and OD_blank2_ is obtained only in the presence of DMEM medium.

The biocompatibility of R‐CDNP was further measured via proteomic perturbation in HepG2 cells. HepG2 cells were incubated in DMEM medium with R‐CDNP (0.6 mg mL^−1^) for 7 h at 37 °C and 5% CO_2_. Then the medium was removed, and cells were washed with PBS three times. Cells without the treatment of R‐CDNP were used as the control. Collected cells were carried out the i‐FASP according to the following protocol.

### In Situ Cross‐Linking of Intracellular Mitochondria Based on R‐CDNP

HepG2 cells were incubated in DMEM medium with R‐CDNP (0.6 mg mL^−1^) for 7 h at 37 °C and 5% CO_2_. Then the medium was removed, and cells were washed with PBS three times. Cells were collected by centrifugation at 500 g for 10 min. Collected cells were carried out the i‐FASP according to the following protocol, and the cross‐linked peptides were further fractionated.

### Sample Preparation by i‐FASP

Collected cells were treated with i‐FASP protocol.^[^ ^40^ ^]^ Cells were dispersed in lysis buffer (10% C12Im‐Cl and 1% protease inhibitor cocktail) followed by 120 W sonication (3 s on, 3 s off, 4 min) in an ice bath. Then the cell debris was removed by centrifugation at 15000 g for 10 min. 1,4‐Dithiothreitol (DTT) (a final concentration of 100 mm) (Sigma‐Aldrich, St Louis, USA) was added to the lysis solution and reacted at 95 °C for 5 min. The denatured protein solution was transferred to a 10 kDa filter and then centrifuged at 15000 g for 20 min. Subsequently, iodoacetamide (IAA) (20 mM) (Sigma‐Aldrich, St Louis, USA) was added for alkylation at room temperature for 30 min in the dark, and the filter was washed three times with ammonium bicarbonate (50 mM) to remove the C12Im‐Cl. The protein was digested with sequencing‐grade trypsin (the ratio of enzyme: protein was 1:25) (Promega, Madison, WI) at 37 °C for 14 h. Afterward, tryptic peptides were collected by centrifugation at 15000 g for 20 min, followed by washing twice with ammonium bicarbonate (10 mM).

### Fractionation of Cross‐Linked Peptides

Cross‐linked peptides were fractionated with high‐pH reversed‐phase HPLC. Mobile phase A was an aqueous solution (pH 10), and mobile phase B consisted of 80% acetonitrile and 20% H_2_O (pH 10). The peptides were loaded onto Durashell C18 column (5 µm, 100 Å, 2.1 mm i.d. × 150 mm) with a flow rate of 0.25 mL min^−1^, and fractionated with 60 min gradient as follows: 2% B for 10 min, 5%–20%B for 20 min, 20%–40% B for 20 min, 40%‐90% B for 5 min, 90% B for 5 min. The fractions were collected every 1 min during 5%‐40% B and collected every 30 s during 40%‐90% B. Finally, all fractions were merged into 20 fractions, and the fractions were dried and dissolved in 0.1% FA.

### LC‐MS/MS Analysis

The peptide samples were analyzed by an Easy‐nano LC 1200 system coupled to an Orbitrap Exploris 480 mass spectrometer with a FAIMS Pro device (Thermo Fisher Scientific, San Jose, CA). Mobile phase A was an aqueous solution containing 0.1% formic acid (FA), and mobile phase B was an acetonitrile solution containing 20% H_2_O and 0.1% FA. Samples were separated by a C18 capillary column (150 µm i.d.× 30 cm; ReproSil‐Pur C18‐AQ particles, 1.9 µm, 120 Å) using a 65 min gradient as follows: 12%–30% B for 45 min, 30%–38% B for 6 min, 38%–95% B for 4 min, 95% B for 10 min at a flow rate of 600 nL min^−1^. FAIMS separations were performed with the following settings: total carrier gas flow was 4 L min^−1^; CV values were −45 and −65 V. MS analysis was performed in a data‐dependent acquisition mode with a resolution of 60 000 (m/z = 200) for full MS scan at a scan range of 350–1500 m/z, and 15 000 for MS/MS scan at an isolation window of 1.6 m/z and a fixed first mass of 110 m/z. The automatic gain control (AGC) target was 3e6 for MS1 scan, and the maximum injection time was 20 ms. Precursors were fragmented by higher‐energy collisional dissociation (HCD) with a normalized collision energy (NCE) of 30%. For label‐free quantification samples of proteomic perturbation experiment, only precursors with charge states 2–8 and intensity over 2.5e4 could be selected for fragmentation. For cross‐linked samples, only precursors with charge states 3–8 and intensity over 2.5e4 could be selected for fragmentation. The scan cycle time between MS and MS/MS was 1 s, and the dynamic exclusion duration was set to 30 s. The AGC target was set to 7.5e4 for MS2 scan, and the maximum injection time was 30 ms.

### Data Analysis and Statistical Analysis

The data was presented as the mean ± standard derivation (DLS) from three experiments. MS raw data of label‐free quantification samples for proteomic perturbation experiment were processed by MaxQuant (version 1.6.1.0) against the UniProt human protein database (downloaded on 2021.12.28).^[^ ^41^ ^]^ The search parameters were as follows: carbamidomethyl of cysteine as a fixed modification, and oxidation of methionine and protein N‐terminal acetylation as variable modifications. Trypsin was set as the enzyme with maximum missed cleavage sites of 2. “Label‐free quantification” was chosen, and “Match between runs” was set with a match time window of 0.7 min and an alignment time window of 20 min. The false discovery rate (FDR) was set to 1% at the peptide spectra match (PSM) level and protein level. The protein intensity of sample group and control group was normalized, and then performed for the Student's *t*‐test by Perseus software (version 1.5.8.5). The proteins in the sample group with an intensity change of over two‐fold and *p *< 0.01 compared to the control group were marked as significantly different proteins.

The mitochondrial protein database was constructed by combining the MitoCarta 3.0 database with the annotated mitochondrial proteins of the UniProt human protein database (downloaded on 2021.12.28).^[^ ^42^ ^]^ MS raw data of cross‐linked samples were processed by pLink 2.0 (version 2.3.9) against the constructed mitochondrial protein database.^[^ ^43^ ^]^ The search parameters were as follows: precursor mass tolerance 20 ppm, fragment mass tolerance 20 ppm, peptide mass 600–6000, peptide length 6–60, carbamidomethyl of cysteine as a fixed modification, and oxidation of methionine and protein N‐terminal acetylation as variable modifications. Cross‐linker was set as DSS (cross‐linked sites K and protein N‐terminus, cross‐link mass shift 138.068, mono‐link mass shift 156.079). Trypsin was set as the enzyme with maximum missed cleavage sites of 3. The FDR was set to 1% at the PSM level, and PSM≥2 was set as the identification threshold for cross‐links.

ComMap was applied to integrate cross‐links with existing structures, further realizing the distance calculation in batch.^[^ ^44^ ^]^ PyMol (Schrodinger LLC, The PyMOL Molecular Graphics System, Version 2.5.0) was used to visualize protein structures where the shortest Cα‐Cα distances of cross‐linked residues were mapped. HDOCK web service was used for protein docking, and PPI networks were visualized by Cytoscape (version 3.9.1).^[^ ^45^, ^46^ ^]^

## Conflict of Interest

The authors declare no conflict of interest.

## Author Contributions

W.Z., K.Y., and L.Z. designed the experiments. W.Z. performed the experiments. W.Z., Y.C., W.F., X.L., Q.Z., B.Z., K.Y., and L.Z. discussed and analyzed the data. W.Z. wrote the manuscript. K.Y. and L.Z. revised the manuscript. Y.Z., K.Y., and L.Z. supervised the research.

## Supporting information



Supporting Information

## Data Availability

The data that support the findings of this study are available in the supplementary material of this article.
