# Supplementary material for: Targeted Analysis of Mitochondrial Protein Conformations and Interactions by Endogenous ROS‐Triggered Cross‐Linker Release
Source: Adv Sci (Weinh). 2024 Oct 30;11(48):2408462. doi: 10.1002/advs.202408462 (PMC11672262; doi:10.1002/advs.202408462)
Supplement: Supplementary file 1 — Supporting Information [file ADVS-11-2408462-s001.docx]

Supporting Information

Targeted Analysis of Mitochondrial Protein Conformations and Interactions by Endogenous ROS-Triggered Crosslinker Release

Wen Zhou, Yuwan Chen, Wenxin Fu, Xinwei Li, Yufei Xia, Qun Zhao, Baofeng Zhao, Yukui Zhang, Kaiguang Yang,* and Lihua Zhang*

*Corresponding author:

[Kaiguang](mailto:xxxxx@xxxx.xxx) Yang (yangkaiguang@dicp.ac.cn);

Lihua Zhang (lihuazhang@dicp.ac.cn)


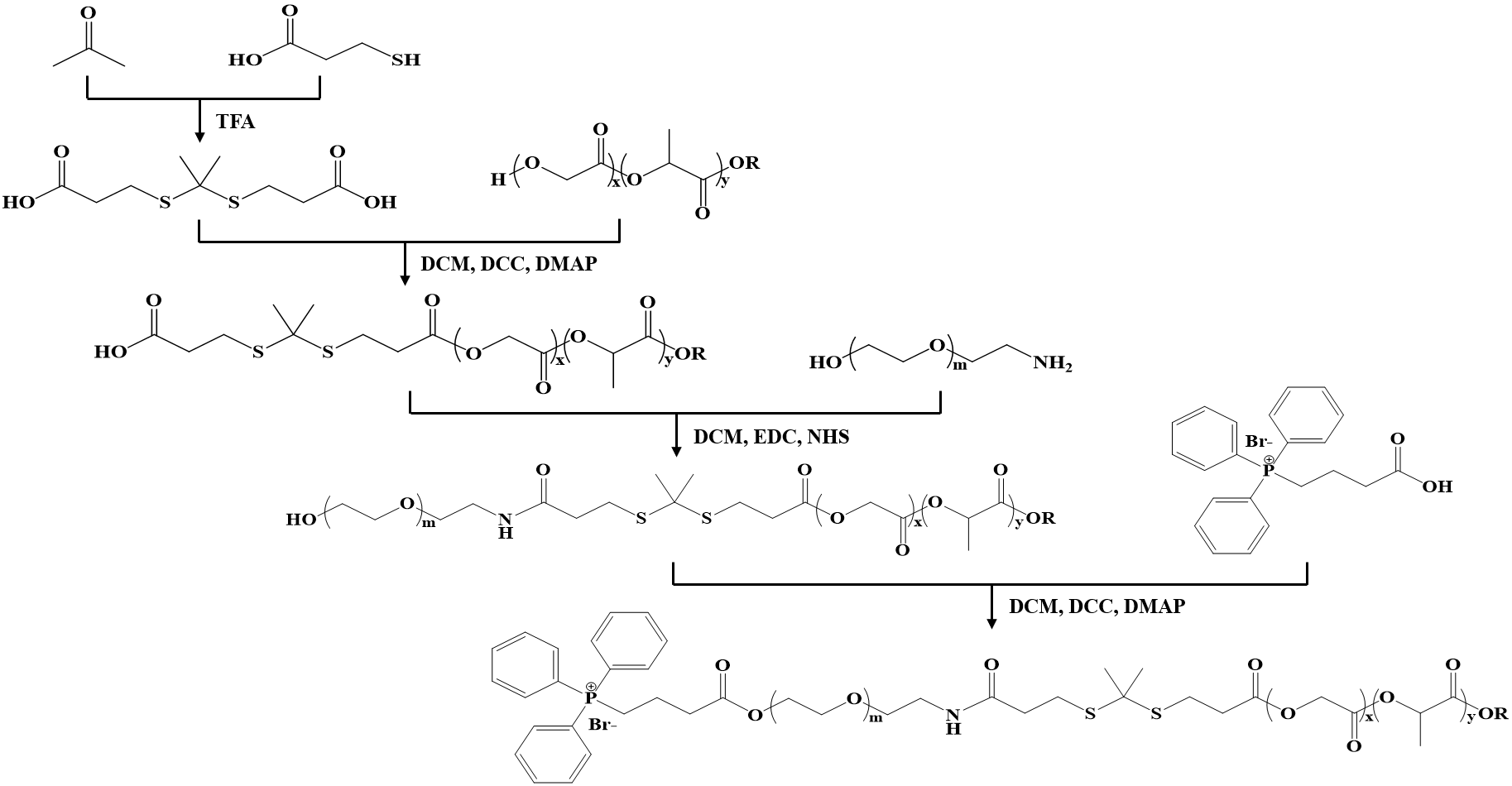


**Figure S1.** Synthesis route of TPP-PEG-TK-PLGA block polymer.


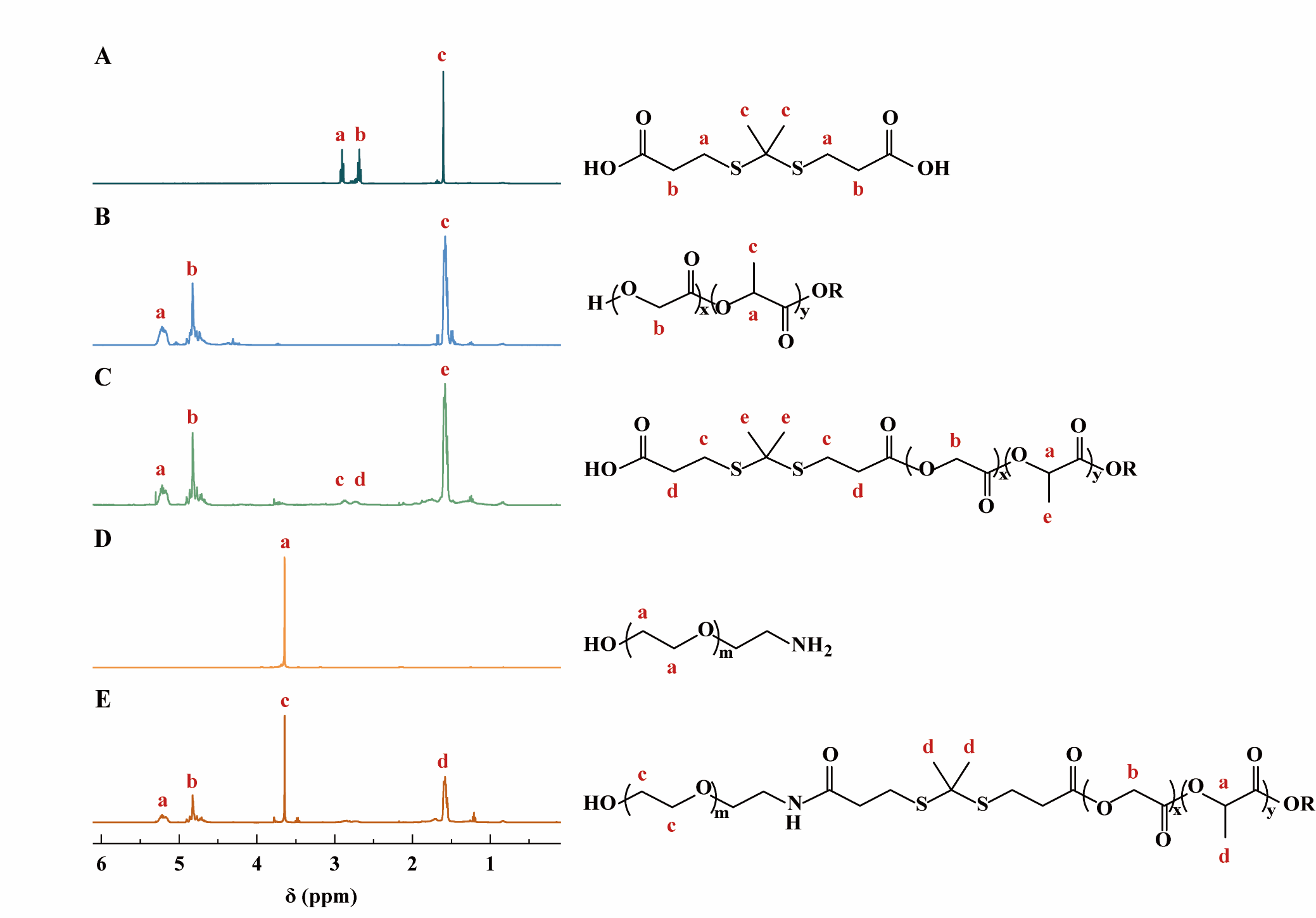


**Figure S2.** ^1^H NMR analysis of intermediates. ^1^H NMR spectra of TK (A), PLGA (B), PLGA-TK (C), PEG (D), and PLGA-TK-PEG (E) in CDCl_3_.


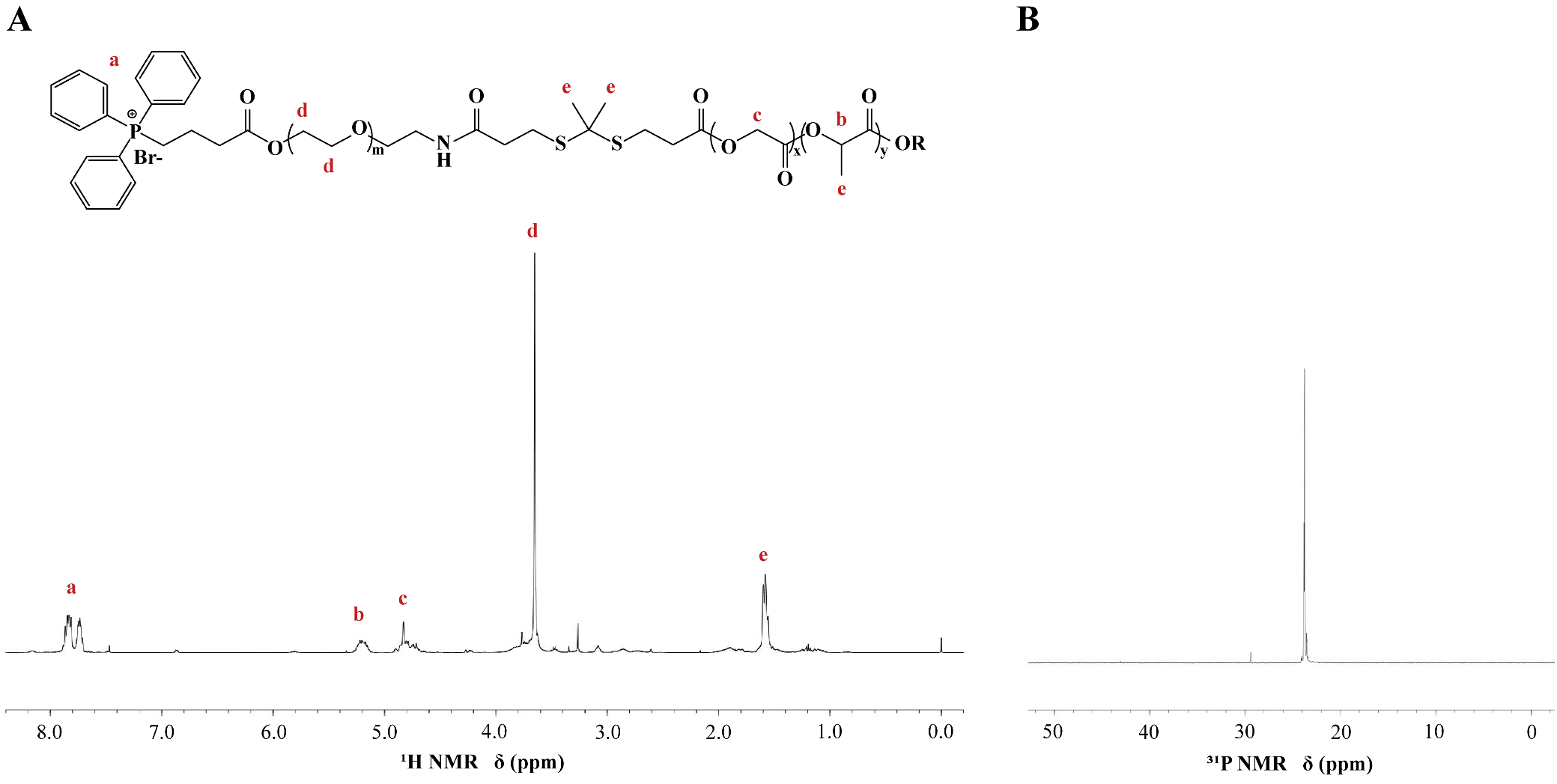


**Figure S3.** NMR analysis of the targeted polymer. (A) ^1^H NMR spectra and (B) ^31^P NMR spectra of TPP-PEG-TK-PLGA polymer in CDCl_3_.


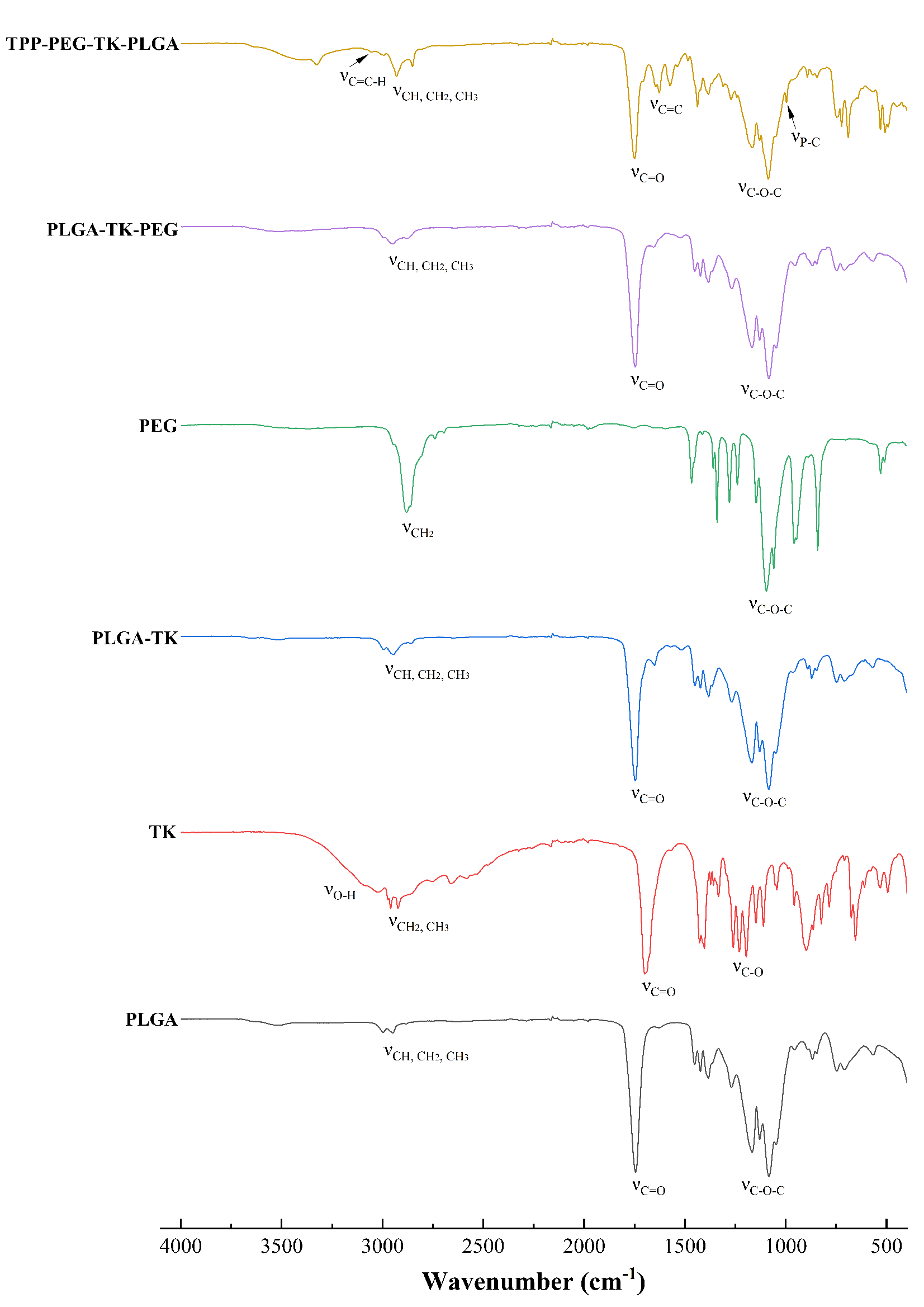


**Figure S4.** FT-IR spectra of polymer materials PLGA and PEG, the intermediates TK, PLGA-TK, and PLGA-TK-PEG, as well as the final copolymer TPP-PEG-TK-PLGA.


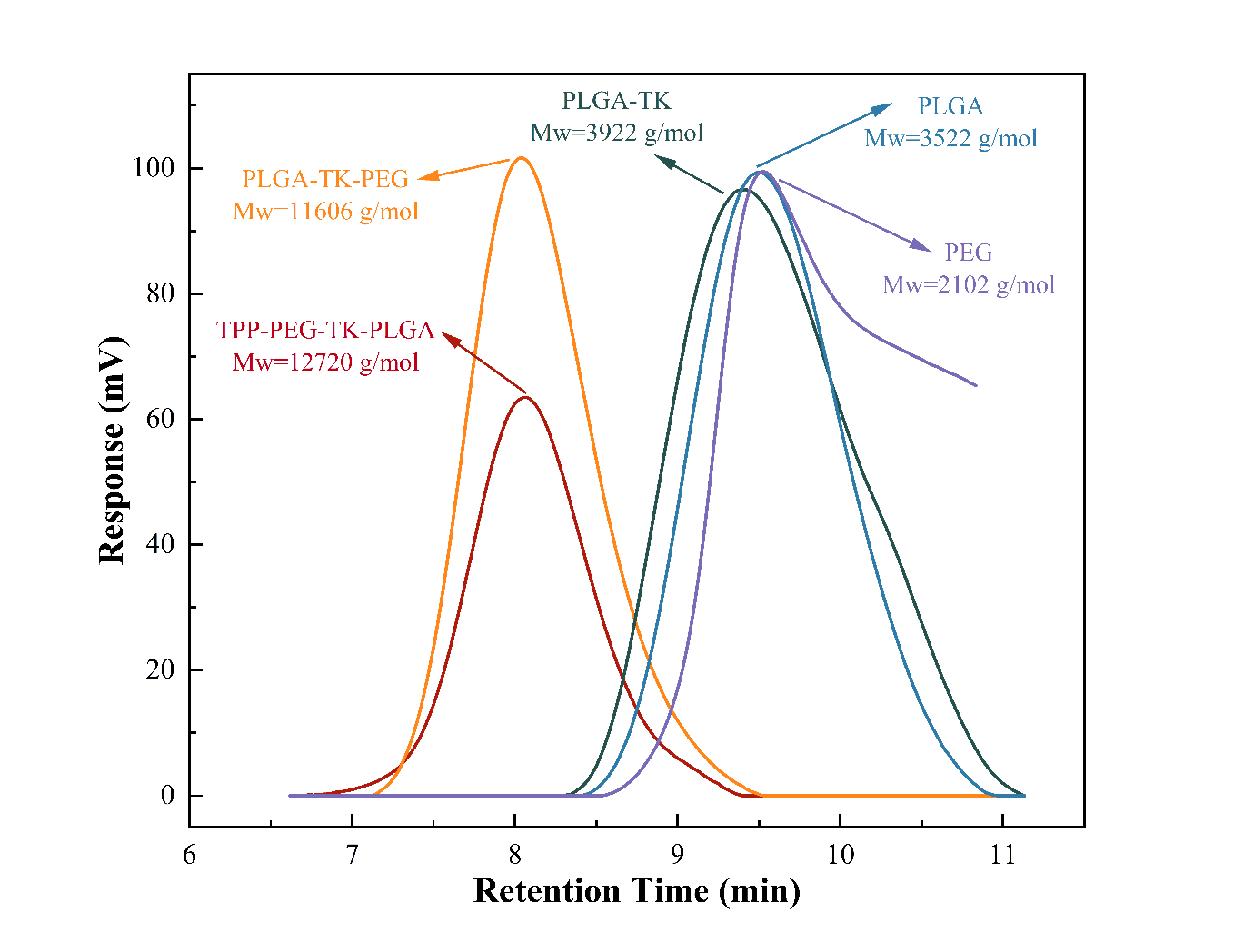


**Figure S5.** GPC chromatograms of polymer materials PLGA and PEG, the intermediates PLGA-TK and PLGA-TK-PEG, as well as the final copolymer TPP-PEG-TK-PLGA.


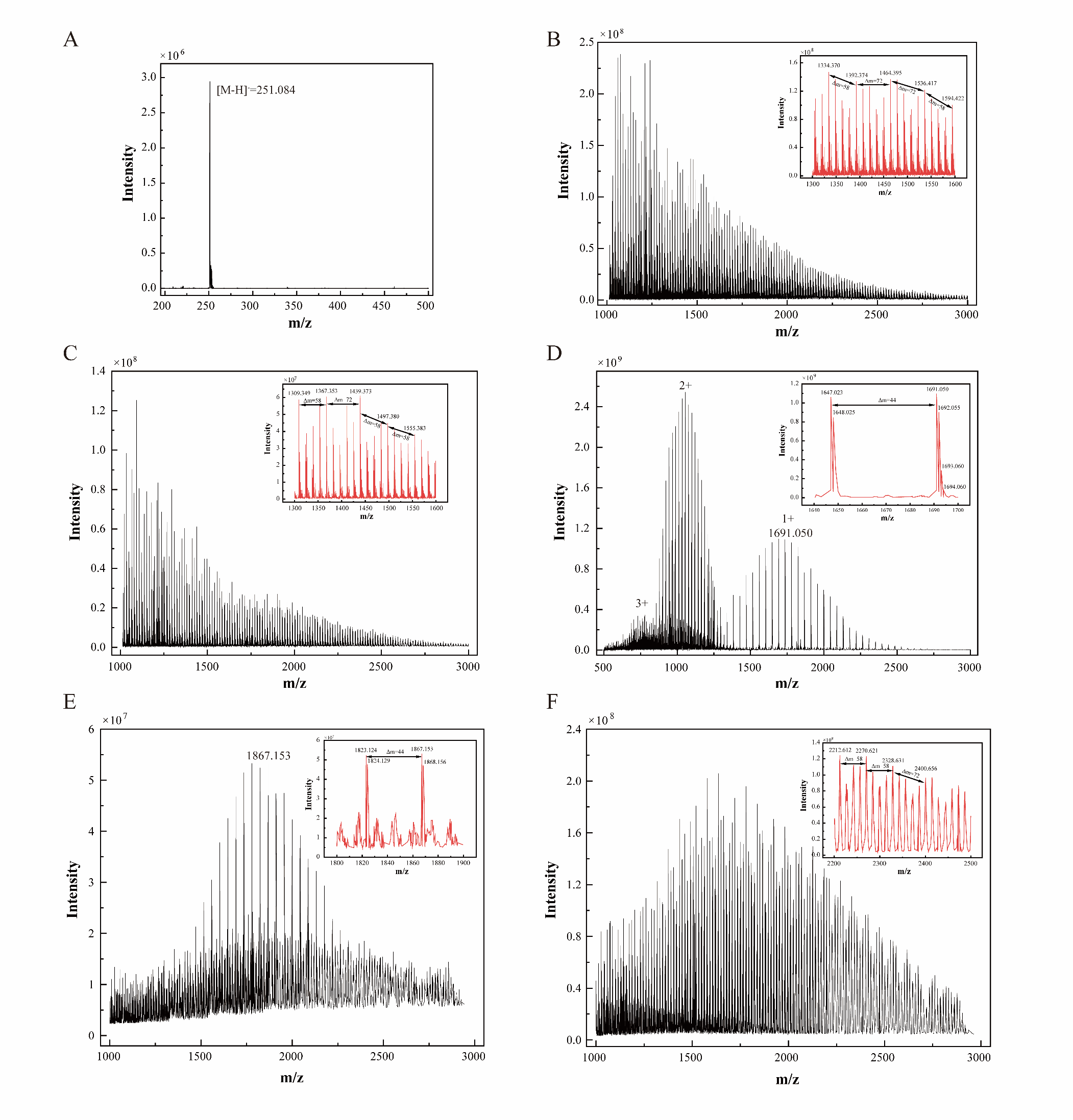


**Figure S6.** ESI spectrum of TK (A), PLGA (B), PLGA-TK (C), PEG (D), PLGA-TK-PEG (E) and TPP-PEG-TK-PLGA (F). The insets showed the identified repeating unit of polymers.


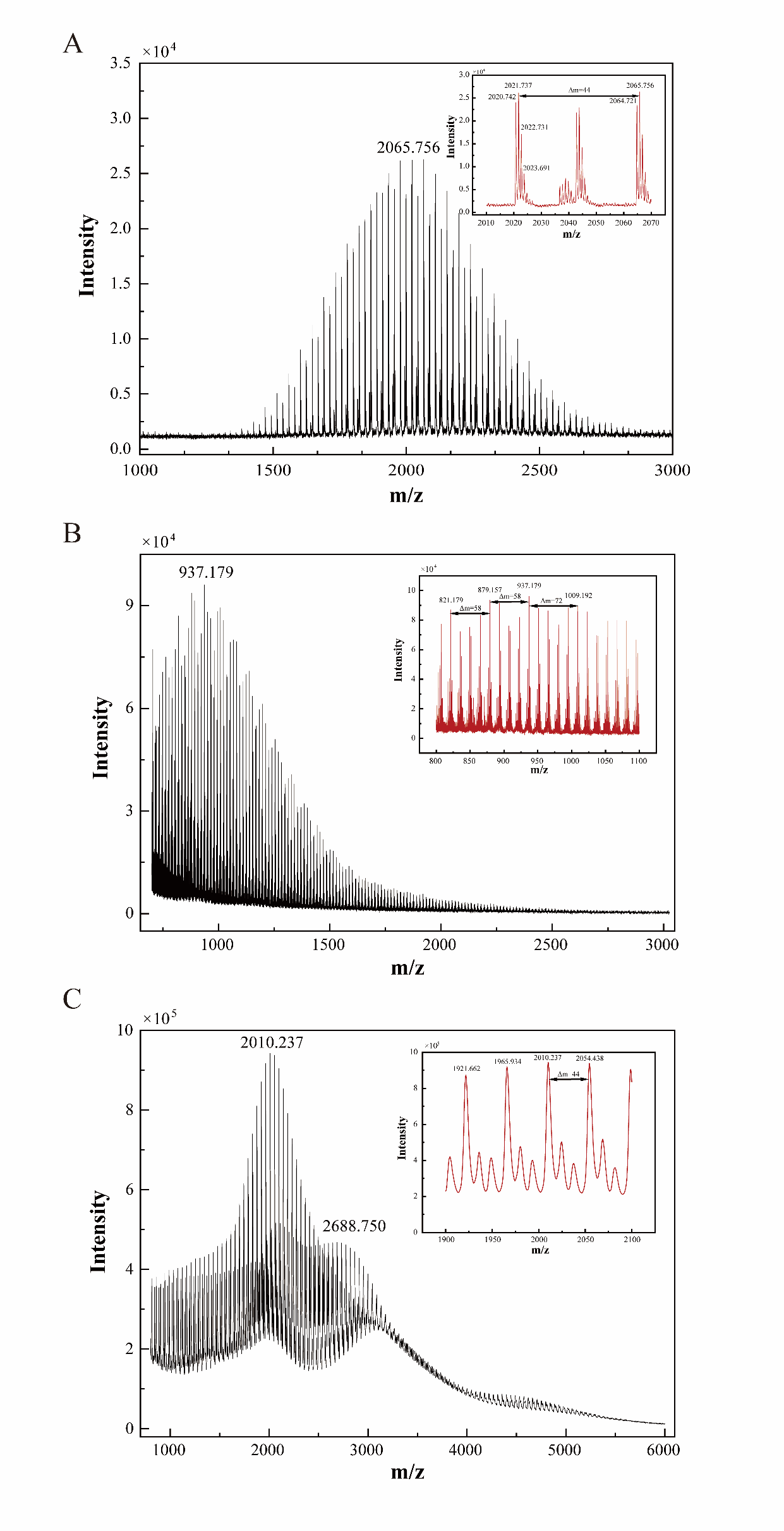


**Figure S7.** MALDI-TOF spectrum of PEG (A), PLGA (B) and TPP-PEG-TK-PLGA (C). The insets showed the identified repeating unit of polymers.


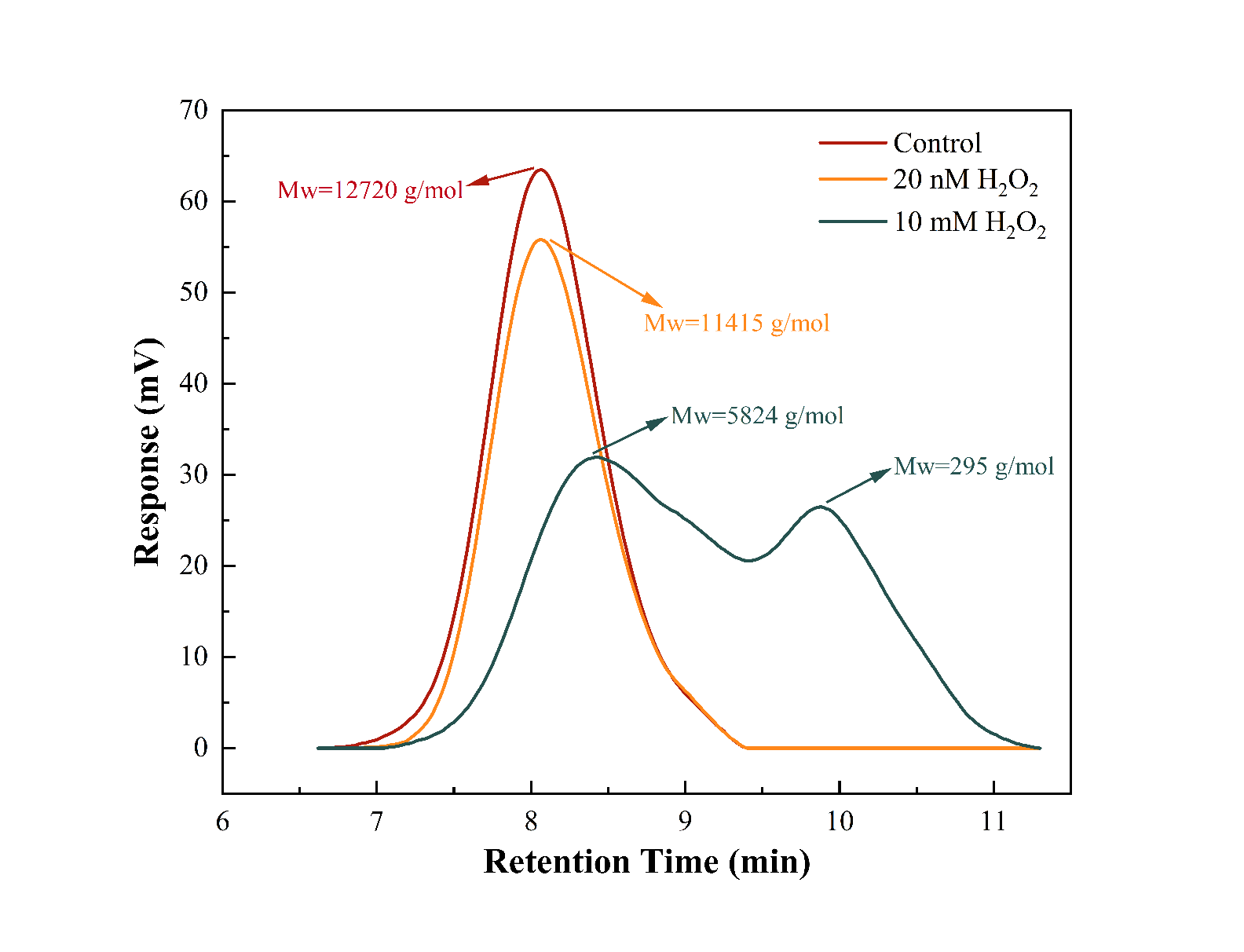


**Figure S8.** GPC profiles of TPP-PEG-TK-PLGA (red line) and the polymers treated with 20 nM H2O2 and 10 mM H2O2 at 37 ℃ for 24 h.


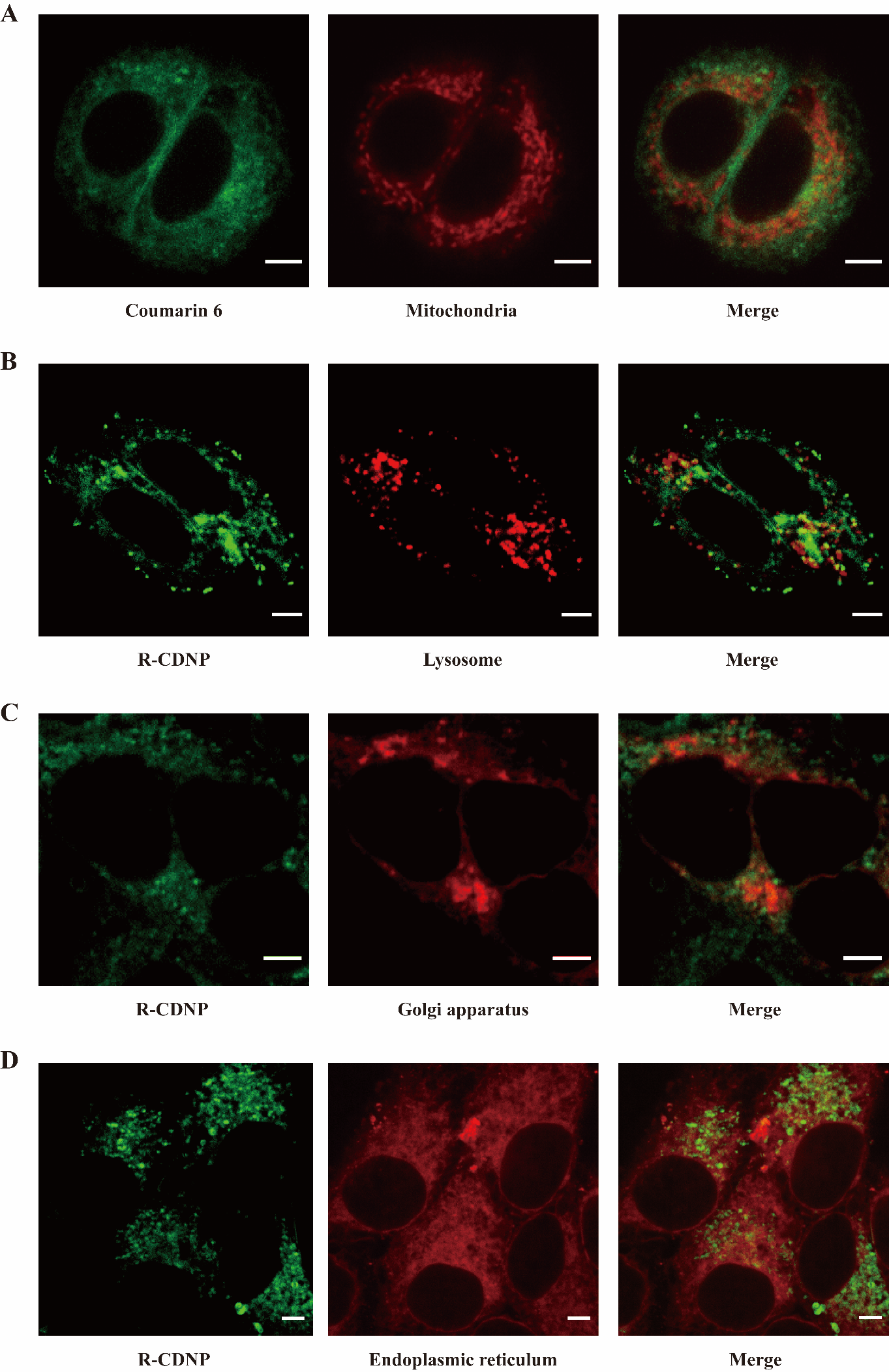


**Figure S9.** Control experiments to characterize the intracellular distributions of R-CDNP. A) CLSM images of HepG2 cells after incubating with Coumarin 6 (green channel) for 3 h. MitoTracker deep red (red channel) was employed to co-stain mitochondria. Scale bars = 5 μm. B) CLSM images of HepG2 cells after incubating with Coumarin 6-labeled R-CDNP for 3 h. Coumarin 6 (green channel) was utilized to label nanoparticles, and LysoTracker Red (red channel) was employed to co-stain lysosome. Scale bars = 5 μm. C) CLSM images of HepG2 cells after incubating with Coumarin 6-labeled R-CDNP for 3 h. Coumarin 6 (green channel) was utilized to label nanoparticles, and Golgi-Tracker Red (red channel) was employed to co-stain Golgi apparatus. Scale bars = 5 μm. D) CLSM images of HepG2 cells after incubating with Coumarin 6-labeled R-CDNP for 3 h. Coumarin 6 (green channel) was utilized to label nanoparticles, and ER-Tracker Red (red channel) was employed to co-stain Endoplasmic reticulum. Scale bars = 5 μm.


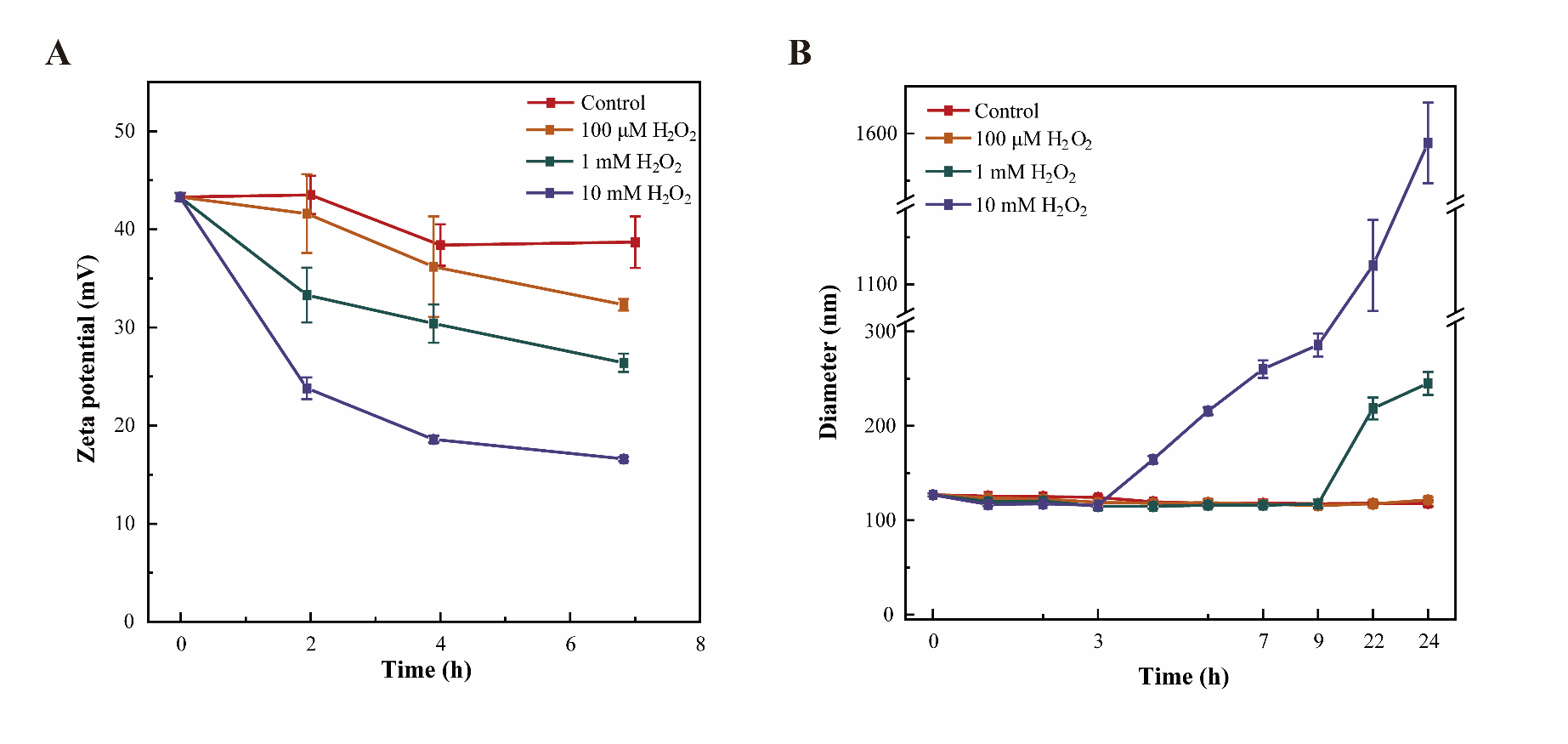


**Figure S10.** DLS characterization of R-CDNP under ROS stimulation. A) The zeta potential changing curves of R-CDNP over time against different H_2_O_2_ concentrations (20 nM, 100 μM, 1 mM, 10 mM) at 37 ℃ (mean ± SD, n = 3 independent experiments). B) The diameter changing curves of R-CDNP over time against different H_2_O_2_ concentrations (20 nM, 100 μM, 1 mM, 10 mM) at 37 ℃ (mean ± SD, n = 3 independent experiments). The general H_2_O_2_ concentration (20 nM) of normal tissues was considered as the control group.


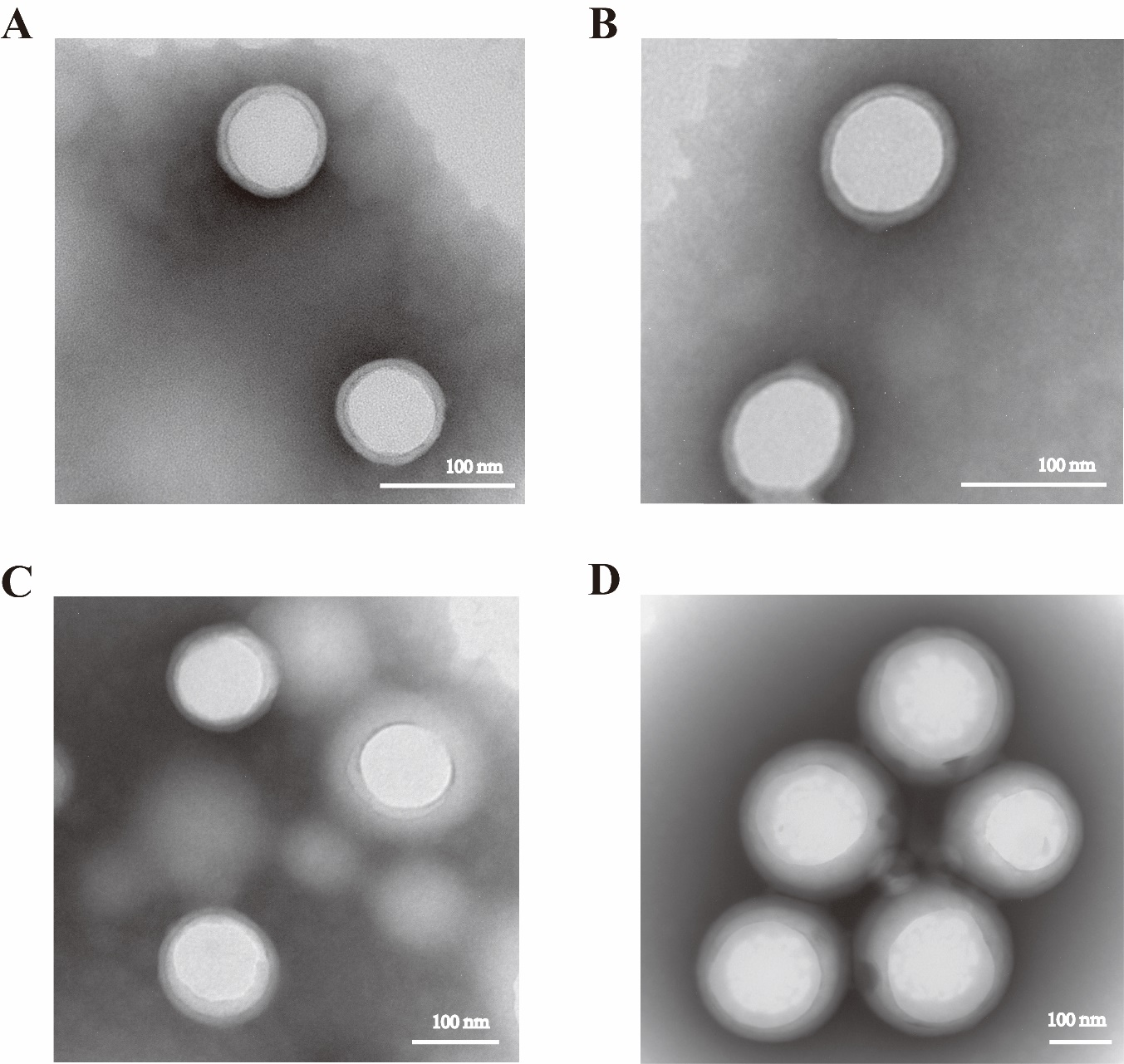


**Figure S11.** TEM images of R-CDNP after 48 h in the absence of H_2_O_2_ (A), in the presence of 20 nM H_2_O_2_ (B), 100 μM H_2_O_2_ (C), and 1 mM H_2_O_2_ (D). The general H_2_O_2_ concentration (20 nM) of normal tissues was considered as the control group. Scale bar = 100 μm.


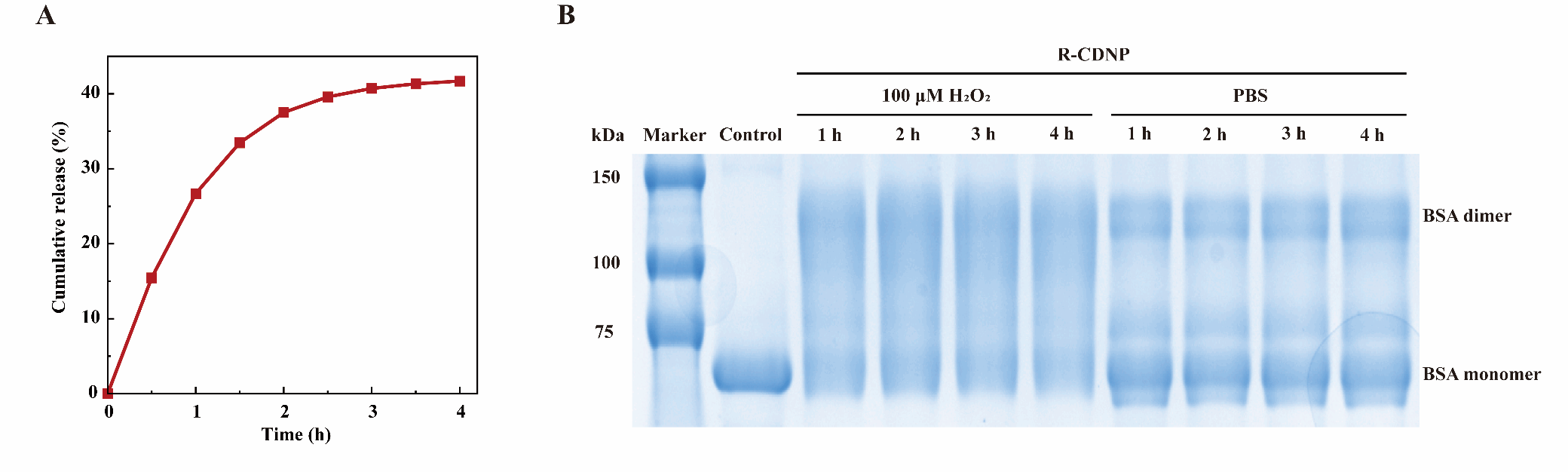


**Figure S12.** ROS-responsiveness of R-CDNP. (A) DSS release kinetics of R-CDNP. R-CDNP was treated with 100 μM H_2_O_2_ at 37 ℃, and released DSS was monitored by HPLC. (B) In vitro crosslinking behaviour of BSA with R-CDNP. BSA was treated with R-CDNP under different environments (100 μM H_2_O_2_ or PBS). SDS-PAGE gels showed the more obvious BSA dimer bonds under 100 μM H_2_O_2_. BSA solution was used as a control.


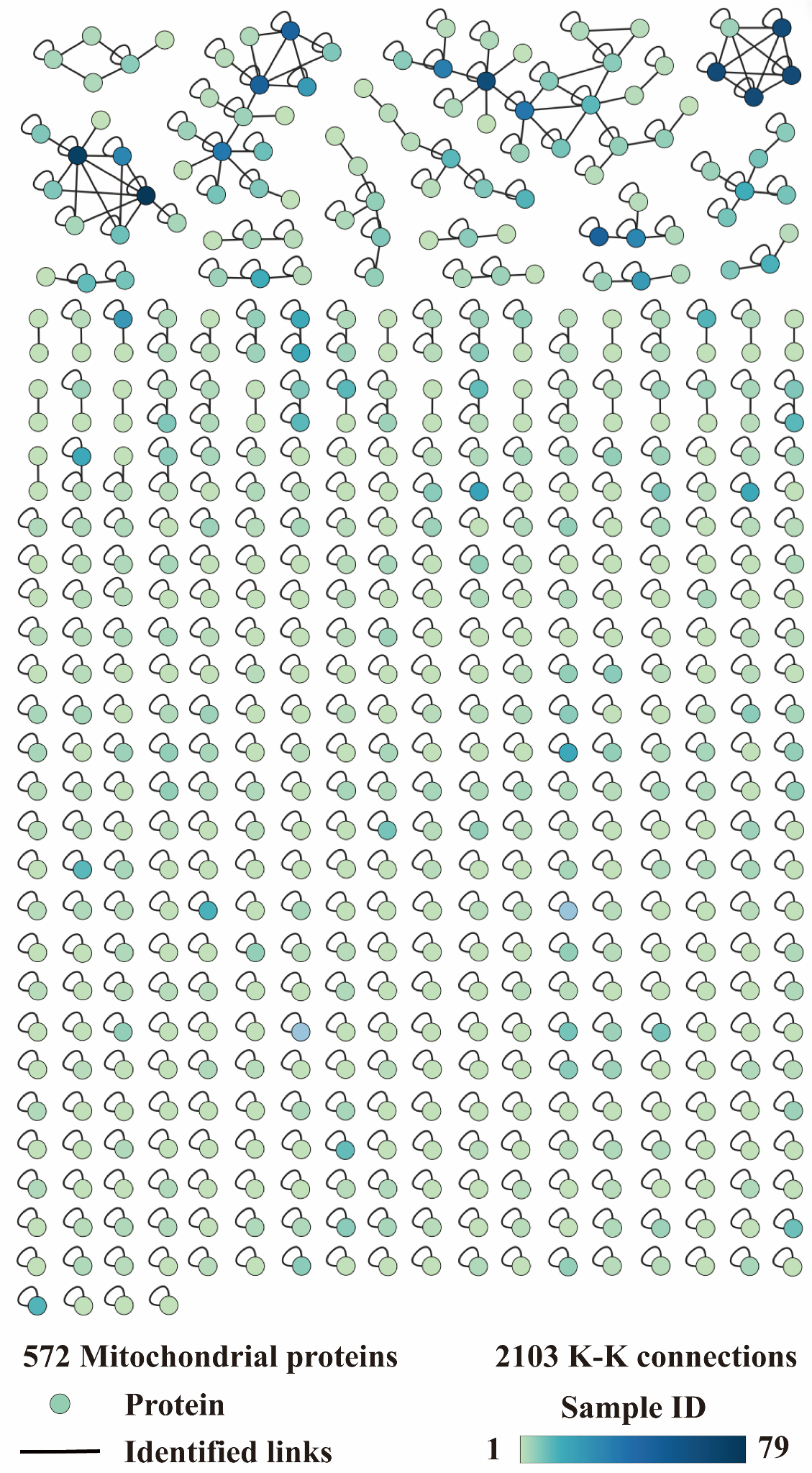


**Figure S13.** The overview of identified mitochondrial protein cross-links. Nodes represented individual proteins; lines represented the identified links (including inter-links between different proteins and intra-links within a protein). Nodes were colored based on the number of identified links.


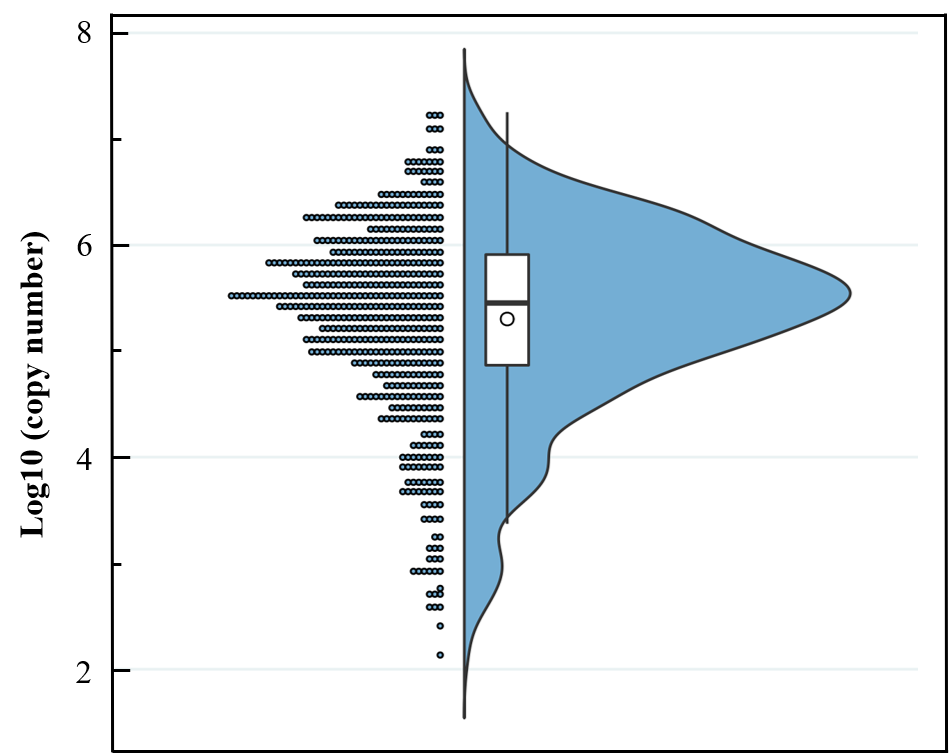


**Figure S14.** The abundance distribution of cross-linked mitochondrial proteins.


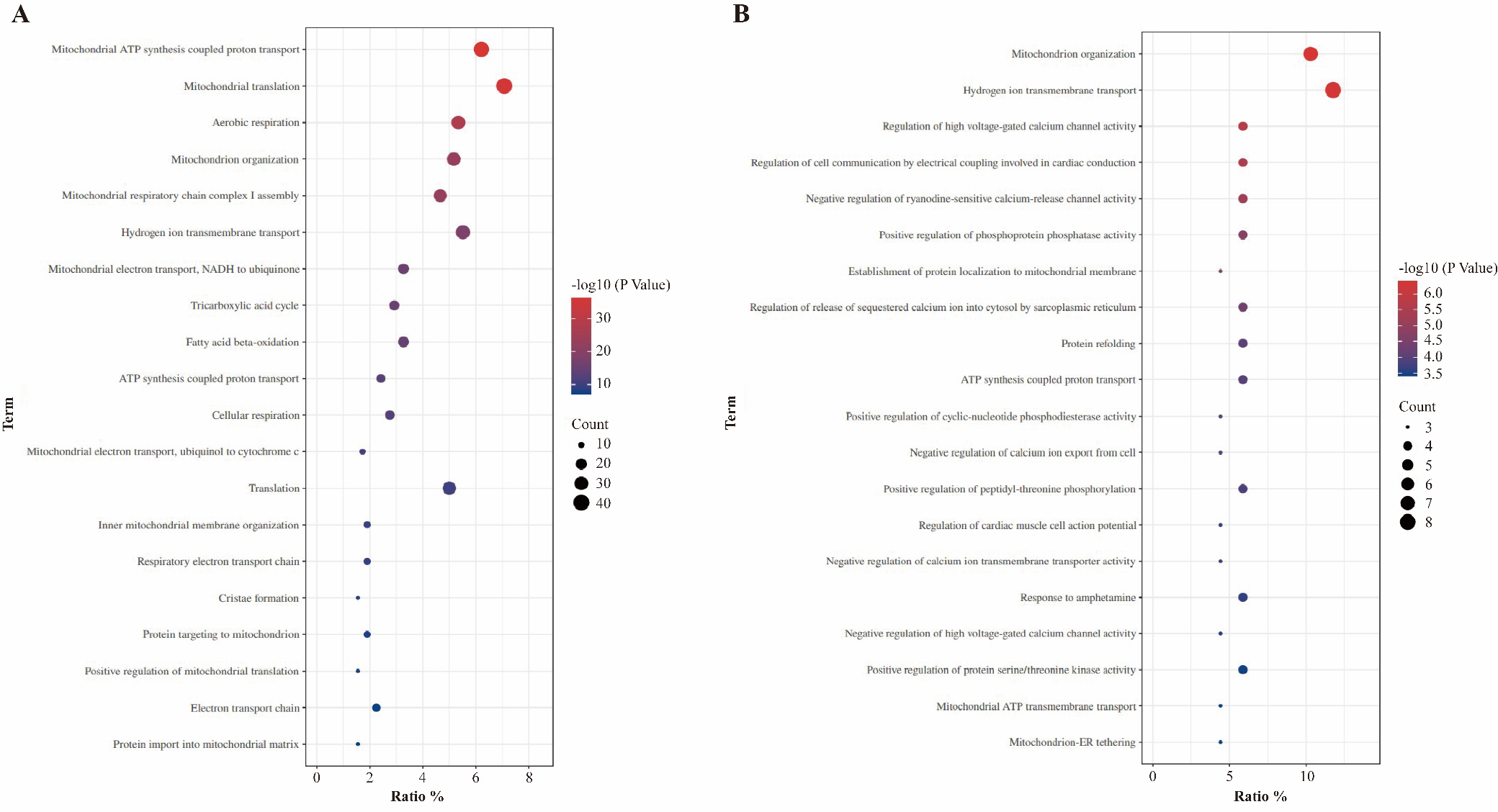


**Figure S15.** GOBP analysis of identified proteins. GOBP analysis of identified 572 mitochondrial proteins (A), and 67 PPIs-related proteins not reported in the STRING databases (B). Node size was related to count, and nodes were colored according to the -log10 (*P* Value) of GOBP analysis.


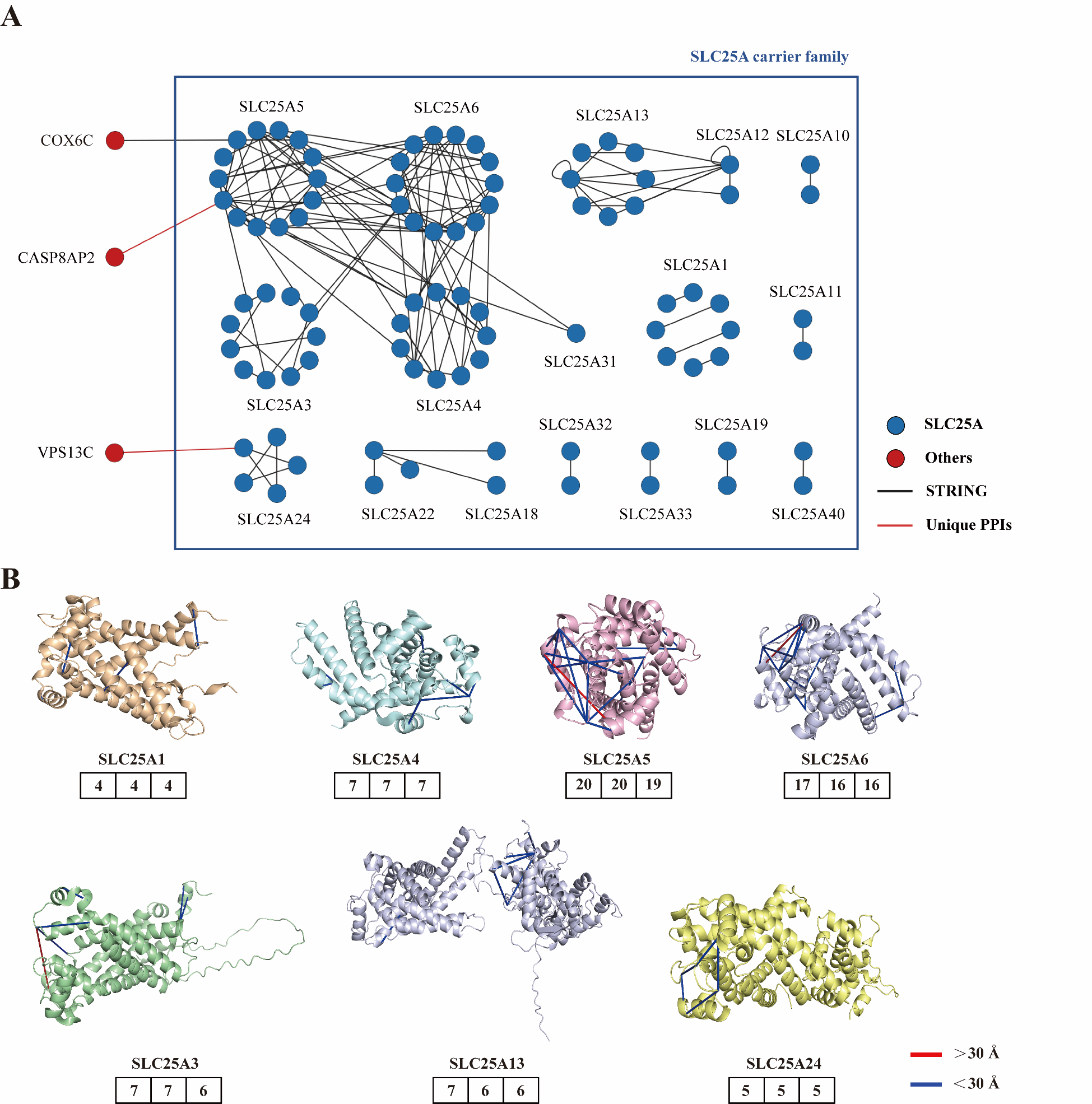


**Figure S16.** The cross-linking information among the SLC25A carrier family. (A) The interaction network. These nodes were indicated using different colors, blue for SLC25A proteins and red for others. The interactions reported in the STRING database were shown in black, and unreported interactions (unique PPIs) were in red. (B) Mapping of identified cross-links onto AlphaFold structures. Blue lines represented the cross-links satisfying the distance restraint, and red lines represented the cross-links exceeding the distance restraint.


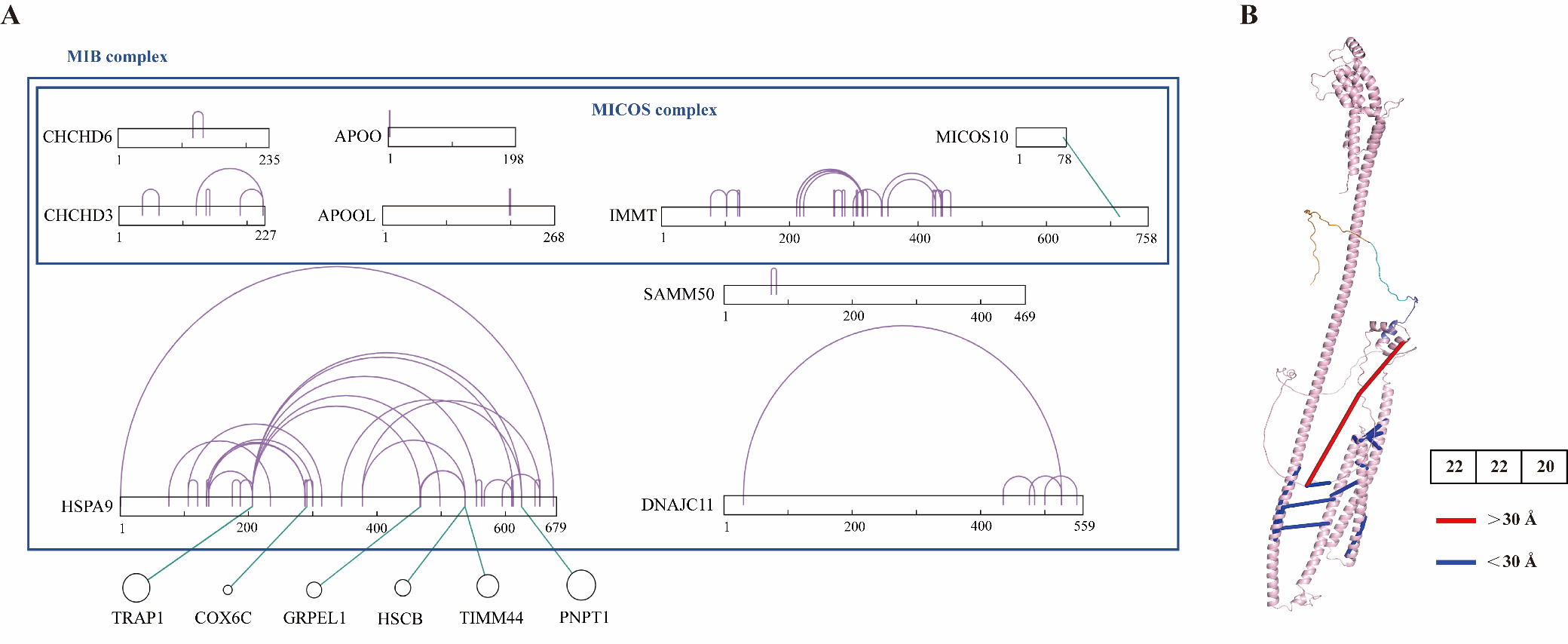


**Figure S17.** The cross-linking information among MIB complex. MIB complex is formed based on the strong interaction between the mitochondrial contact site and cristae organizing system complex (MICOS) of IMM and the sorting and assembly machinery complex (SAM) of OMM. (A) The interaction network of MIB complex. (B) Mapping of identified cross-links onto AlphaFold structure of IMMT, a protein subunit of MIB complex. Blue lines represented the cross-links satisfying the distance restraint, and red lines represented the cross-links exceeding the distance restraint.


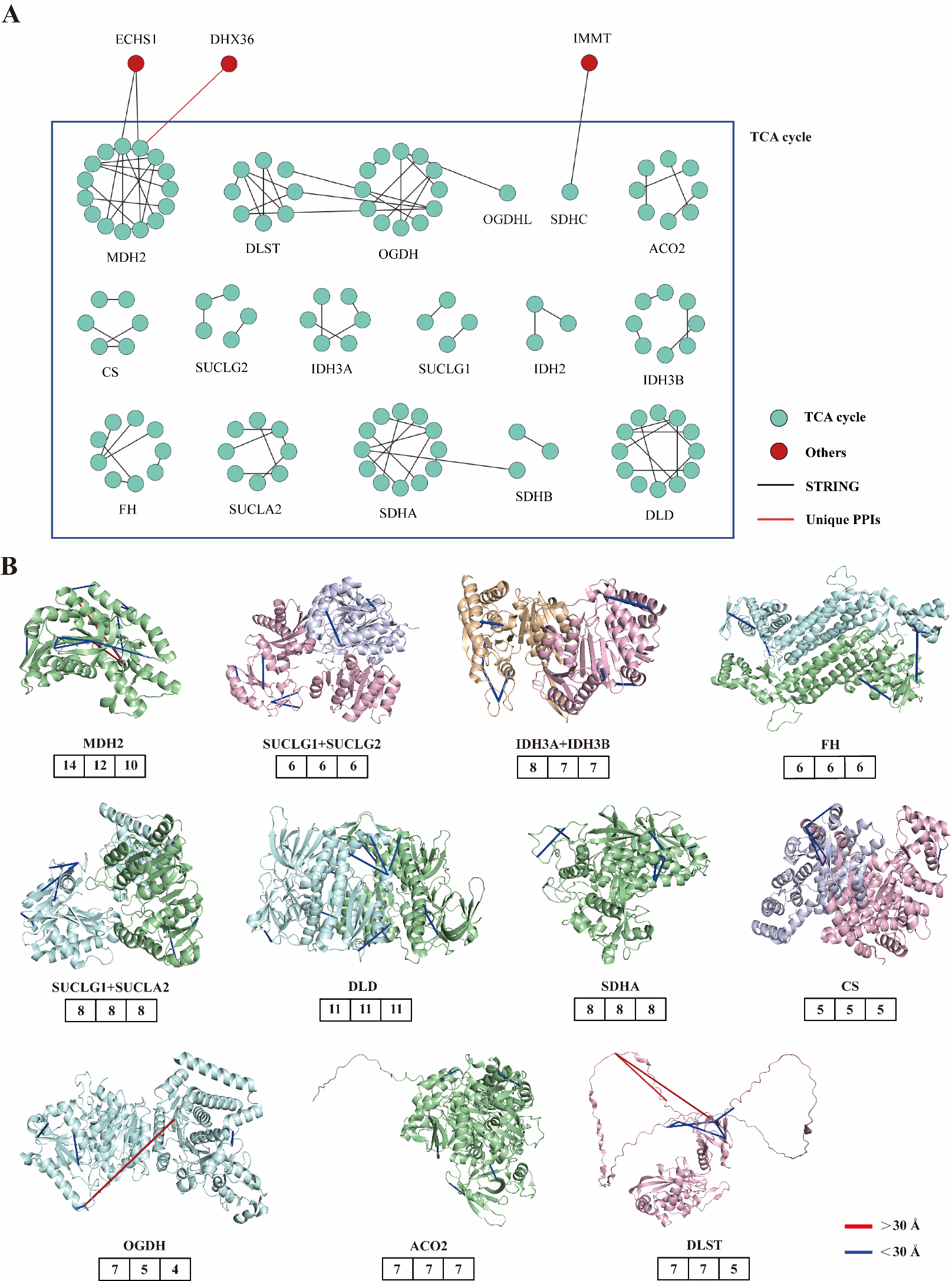


**Figure S18.** The cross-linking information of TCA cycle. (A) The interaction network. These nodes were indicated using different colors, green for TCA cycle proteins and red for others. The interactions reported in the STRING database were shown in black, and unreported interactions (unique PPIs) were in red. (B) Mapping identified cross-links onto PDB structures of MDH2 (PDB: 4WLE, only A chain), SUCLG1+SUCLG2 (PDB: 6WCV), IDH3A+IDH3B (PDB: 6KDE, only A and B chain), FH (PDB: 5UPP, only A and B chain), SUCLG1+SUCLA2 (PDB: 6G4Q), DLD (PDB: 6I4R), SDHA (PDB: 6VAX), CS (PDB: 5UZR), OGDH (PDB: 7WGR, only A chain), and AlphaFold structures of ACO2 and DLST. Blue lines represented the cross-links satisfying the distance restraint, and red lines represented the cross-links exceeding the distance restraint.


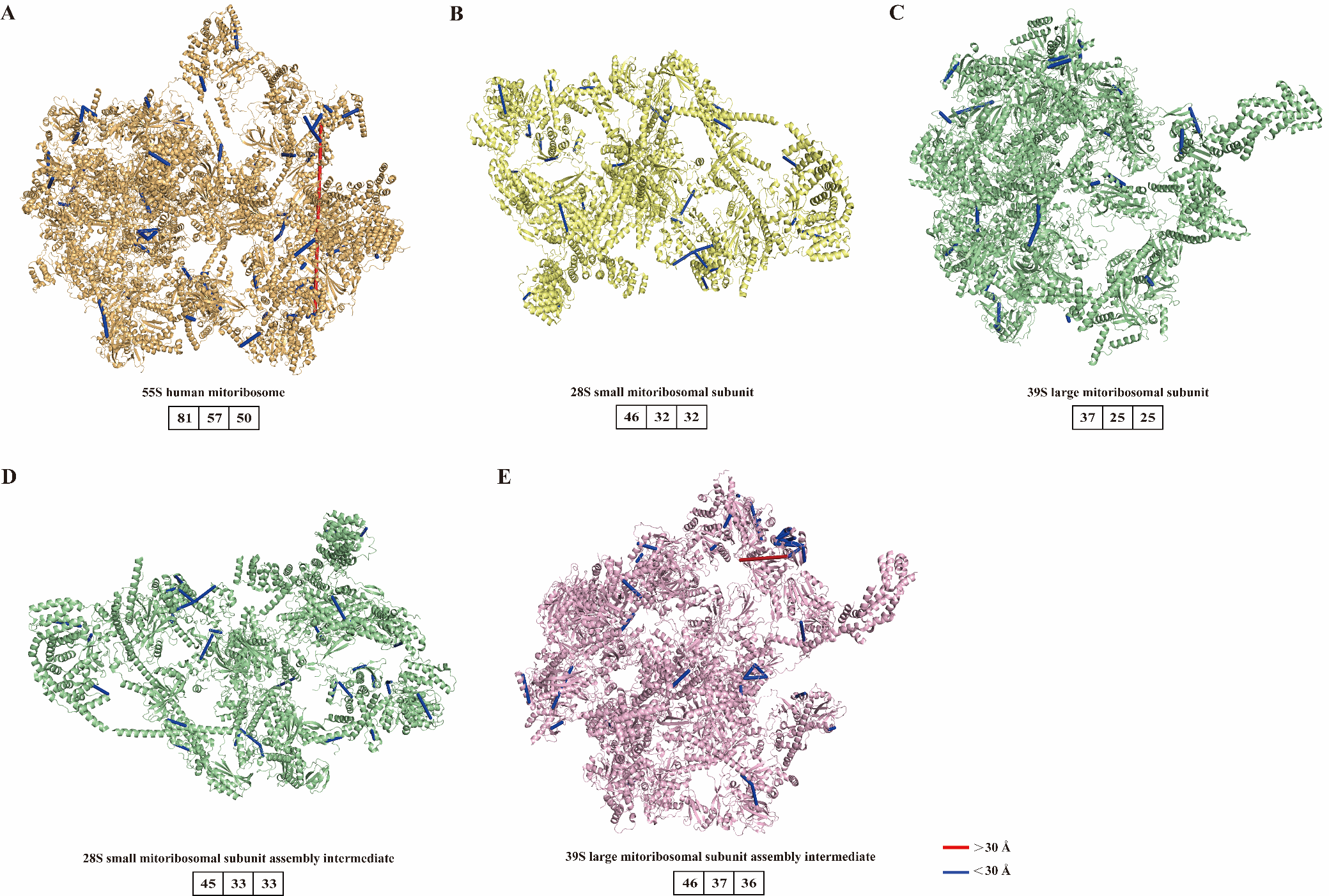


**Figure S19.** The cross-linking information among human mitoribosome. 55S human mitoribosome consists of a 28S small mitoribosomal subunit and a 39S large mitoribosomal subunit, and stable assembly intermediates are formed during the assembly of mitoribosomal subunits. (A), (B), (C), (D), and (E) Mapping of identified cross-links onto the protein structures of 55S mitoribosome (PDB: 8OIR), 28S human small mitoribosomal subunit (PDB: 8OIS), 39S human large mitoribosomal subunit (PDB: 8OIT), 28S human small mitoribosomal subunit assembly intermediate (PDB: 7PO0), and 39S human large mitoribosomal subunit assembly intermediate (PDB: 7O9K). Blue lines represented the cross-links satisfying the distance restraint, and red lines represented the cross-links exceeding the distance restraint.


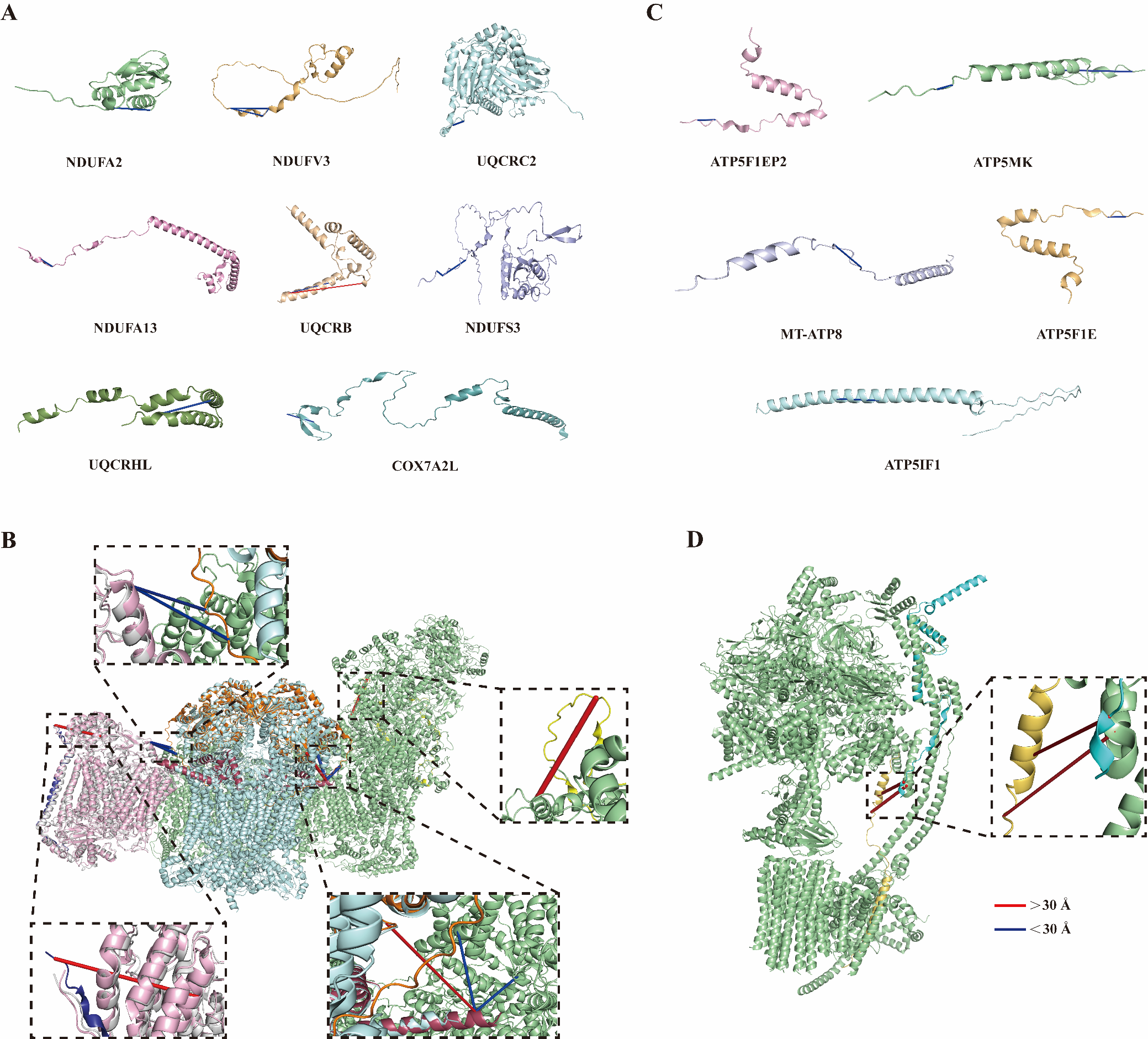


**Figure S20.** The cross-linking information that could not be mapped onto the crystal structures of OXPHOS complexes. (A) 12 intra-links which failed to be mapped on the crystal structure of CⅠ-CⅢ_2_-CⅣ (PDB: 5XTH + 5Z62) were mapped on AlphaFold structures. (B) 7 inter-links were mapped on combined structures. AlphaFold structures of UQCRC2 (shown in orange), UQCRB (shown in raspberry), COX6C (shown in deepblue) and NDUFA7 (shown in yellow) were used to supplement the missing part of CⅠ-CⅢ_2_-CⅣ crystal structure (PDB: 5XTH + 5Z62). CⅠ, CⅢ, and CⅣ of PDB 5XTH were in palegreen, palecyan, and gray. CⅣ of PDB 5Z62 was in lightpink. (C) 6 intra-links which failed to be mapped on the crystal structure of CⅤ (PDB: 6ZQM) were mapped on AlphaFold structures. (D) 2 inter-links were mapped on combined structures. AlphaFold structures of MT-ATP8 (shown in yelloworange) and ATP5PF (shown in cyan) were used to supplement the missing part of CⅤ crystal structure (PDB: 6ZQM shown in palegreen). Blue lines represented the cross-links satisfying the distance restraint, and red lines represented the cross-links exceeding the distance restraint.


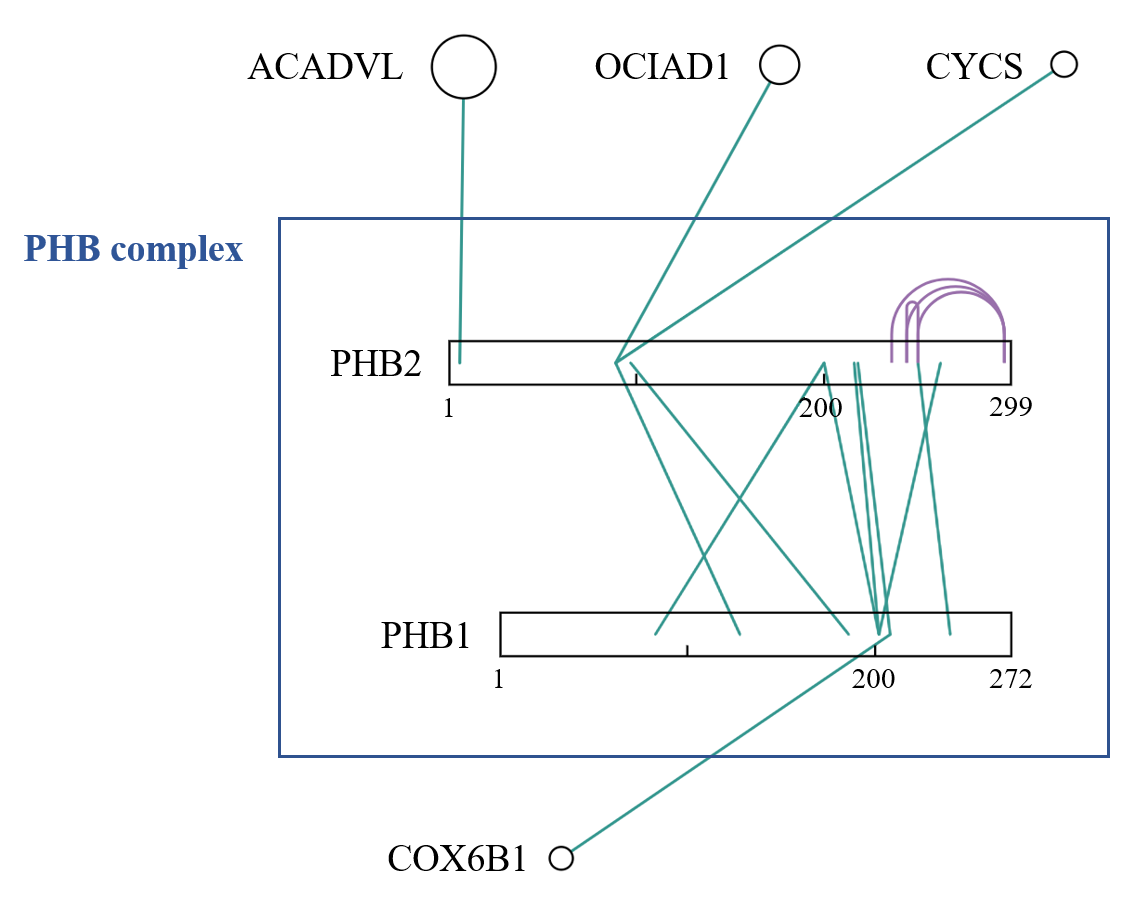


**Figure S21.** The interaction network for PHB complex.

**Table S1.** The optimization for preparing DSS-loaded nanoparticles.

| **Polymer** | **Polymer concentration** | **Polymer: DSS** | **Oil phase** | **Internal water phase** | **External water phase** | **Ultrasonic power / time** | **Size**  **(nm)** | **PDI** | **Zeta potential (mV)** | **Loading efficiency** |
| --- | --- | --- | --- | --- | --- | --- | --- | --- | --- | --- |
| TPP-PEG-TK-PLGA | 50 mg/mL | 10:1 | DCM | H_2_O | H_2_O | 120 W / 4 min | 124.2 | 0.408 | 54.2 | 0.70% |
|  |  | 5:1 |  |  |  |  | 118.4 | 0.395 | 51.7 | 1.75% |
| TPP-PEG-TK-PLGA | 50 mg/mL | 5:1 | DCM | H_2_O | H_2_O | 120 W / 4 min | 118.4 | 0.395 | 51.7 | 1.75% |
|  |  |  |  |  |  | 80 W / 4 min | 113.6 | 0.375 | 57.5 | 1.55% |
|  |  |  |  |  |  | 120 W / 2 min | 102.0 | 0.274 | 51.8 | 0.92% |
| TPP-PEG-TK-PLGA | 20 mg/mL | 5:1 | DCM | H_2_O | H_2_O | 120 W / 2 min | 159.0 | 0.169 | 31.6 | 0.42% |
|  | 50 mg/mL |  |  |  |  |  | 150.2 | 0.185 | 36.2 | 0.94% |
|  | 80 mg/mL |  |  |  |  |  | 243.8 | 0.214 | 37.7 | 0.62% |
| TPP-PEG-TK-PLGA | 50 mg/mL | 5:1 | DCM | H_2_O | H_2_O | 120 W / 2 min | 181.2 | 0.211 | 30.8 | 0.36% |
| 80% TPP-PEG-TK-PLGA + 20% PLGA |  |  |  |  |  |  | 255.1 | 0.169 | 21.7 | 0.78% |
| 80% TPP-PEG-TK-PLGA + 20% PLGA | 50 mg/mL | 5:1 | DCM | H_2_O | H_2_O | 120 W / 2 min | 255.1 | 0.169 | 21.7 | 0.78% |
|  |  |  | DCM: AC=4:1 |  |  |  | 226.5 | 0.163 | 12.9 | 0.38% |
| 80% TPP-PEG-TK-PLGA + 20% PLGA | 50 mg/mL | 5:1 | DCM | H_2_O | H_2_O | 120 W / 2 min | 182.5 | 0.279 | 22.6 | 1.29% |
|  |  |  |  |  | EtOH: H_2_O=9:1 |  | 1107 | 0.238 | 23.2 | 0.77% |
|  |  |  |  | 1% PVA | EtOH: 0.4% PVA=6:4 |  | 265.3 | 0.226 | -5.67 | 13.01% |
|  |  |  |  |  | EtOH: 0.4% PVA=4:6 |  | 232.3 | 0.079 | -12.0 | 1.27% |
|  |  |  |  |  | EtOH: 0.4% PVA=2:8 |  | 205.2 | 0.375 | -13.4 | 1.07% |
| 80% TPP-PEG-TK-PLGA + 20% PLGA | 50 mg/mL | 5:1 | DCM | 1% PVA | EtOH: 0.4% PVA=6:4 | 120 W / 2 min | 265.3 | 0.226 | -5.67 | 13.01% |
|  |  |  |  | 1% F68 | EtOH: 0.4% F68=6:4 |  | 146.6 | 0.187 | 31.4 | 8.75% |

**Table S2.** The similarities of CC, MF and BP of newly identified interacting proteins. The threshold for potential interaction was set as similarities ≥ 0.4.

| **Protein A** | **Protein B** | **Gene A** | **Gene B** | **Similarity of CC** | **Similarity of MF** | **Similarity of BP** |
| --- | --- | --- | --- | --- | --- | --- |
| Q5T2N8 | Q9NVI7 | ATAD3C | ATAD3A | 1 | 0.834 | 0.834 |
| P40926 | Q9H2U1 | MDH2 | DHX36 | 0.748 | 0.535 | 0.535 |
| P40939 | P56385 | HADHA | ATP5ME | 0.832 | 0.343 | 0.343 |
| P10809 | Q8WV93 | HSPD1 | AFG1L | 0.579 | 0.631 | 0.631 |
| Q9BW91 | Q9NRZ5 | NUDT9 | AGPAT4 | 0.56 | 0.464 | 0.464 |
| P36542 | Q5VTU8 | ATP5F1C | ATP5F1EP2 | 0.908 | 0.553 | 0.553 |
| O75521 | P48047 | ECI2 | ATP5PO | 0.686 | 0.5 | 0.5 |
| P10809 | Q7L592 | HSPD1 | NDUFAF7 | 0.58 | 0.526 | 0.526 |
| P0DMV8 | P0DMV9 | HSPA1A | HSPA1B | 0.996 | 0.916 | 0.916 |
| P0DP23 | P0DP25 | CALM1 | CALM3 | 0.97 | 0.94 | 0.94 |
| P08238 | Q58FF3 | HSP90AB1 | HSP90B2P | 0.62 | 0.637 | 0.637 |
| B7ZC32 | P08238 | KIF28P | HSP90AB1 | 0.349 | 0.413 | 0.413 |
| P08574 | P0C7P4 | CYC1 | UQCRFS1P1 | 1 | 0.696 | 0.696 |
| P49748 | Q99623 | ACADVL | PHB2 | 0.585 | 0.554 | 0.554 |
| P09669 | Q16822 | COX6C | PCK2 | 0.721 | 1 | 1 |
| P13995 | Q5JRX3 | MTHFD2 | PITRM1 | 0.851 | 0.279 | 0.279 |
| P04181 | Q9Y6H3 | OAT | ATP23 | 0.715 | 0.456 | 0.456 |
| P04181 | Q86UV5 | OAT | USP48 | 0.892 | 0.431 | 0.431 |
| Q00005 | Q8TCS8 | PPP2R2B | PNPT1 | 0.717 | 0.424 | 0.424 |
| P10809 | Q96AQ8 | HSPD1 | MCUR1 | 0.475 | 1 | 1 |
| P0CG47 | Q15398 | UBB | DLGAP5 | 0.594 | 0.69 | 0.69 |
| P62979 | Q15398 | RPS27A | DLGAP5 | 0.585 | 0.545 | 0.545 |
| Q8TC71 | Q96PN6 | SPATA18 | ADCY10 | 0.616 | 0.384 | 0.384 |
| P82933 | Q9Y3M8 | MRPS9 | STARD13 | 0.631 | 0.535 | 0.535 |
| P05141 | Q9UKL3 | SLC25A5 | CASP8AP2 | 0.687 | 0.351 | 0.351 |
| Q6NUK1 | Q709C8 | SLC25A24 | VPS13C | 0.558 | - | - |
| Q70CQ3 | Q9H583 | USP30 | HEATR1 | 0.577 | 0.427 | 0.427 |
| P39210 | Q9UGM6 | MPV17 | WARS2 | 0.665 | 0.062 | 0.062 |
| P0C7P4 | Q5U5X0 | UQCRFS1P1 | LYRM7 | 0.581 | 0.316 | 0.316 |
| P49588 | Q9NWR8 | AARS1 | MCUB | 0.697 | 0.048 | 0.048 |
| P49792 | Q15311 | RANBP2 | RALBP1 | 0.707 | 0.494 | 0.494 |
| Q8WV60 | Q9BRX8 | PTCD2 | PRXL2A | 0.619 | 0.306 | 0.306 |
| O14786 | Q8NE62 | NRP1 | CHDH | 0.877 | 0.463 | 0.463 |
| P10809 | Q01831 | HSPD1 | XPC | 0.658 | 0.566 | 0.566 |
| O95202 | P48723 | LETM1 | HSPA13 | 0.585 | 0.412 | 0.412 |
| P30626 | Q9UIF7 | SRI | MUTYH | 0.549 | 0.524 | 0.524 |
| A0A0B4J2D5 | P0DPI2 | GATD3B | GATD3 | - | - | - |
